# Gymnastic-Based Movement Therapy for Children With Neurodevelopmental Disabilities: Results From a Pilot Feasibility Study

**DOI:** 10.3389/fped.2019.00186

**Published:** 2019-05-14

**Authors:** Mojgan Gitimoghaddam, William H. McKellin, Anton R. Miller, Jonathan A. Weiss, Annette Majnemer, Louise C. Mâsse, Rollin Brant, Vivien Symington, Robert L. Wishart, Jean-Paul Collet

**Affiliations:** ^1^Department of Pediatrics, University of British Columbia, Vancouver, BC, Canada; ^2^BC Children's Hospital Research Institute, Vancouver, BC, Canada; ^3^Department of Anthropology, University of British Columbia, Vancouver, BC, Canada; ^4^Sunny Hill Health Centre for Children, Vancouver, BC, Canada; ^5^Department of Psychology, York University, Toronto, ON, Canada; ^6^School of Physical and Occupational Therapy, McGill University, Montreal, QC, Canada; ^7^School of Population and Public Health, University of British Columbia, Vancouver, BC, Canada; ^8^Department of Statistics, University of British Columbia, Vancouver, BC, Canada; ^9^Club Aviva Recreation Ltd., Coquitlam, BC, Canada

**Keywords:** physical activity, movement therapy, children, neurodevelopment disability, child development, gymnastic, motor skill, activity theory

## Abstract

**Background:** Developmental and behavioral issues often limit the participation of children with neurodevelopmental disabilities (NDD) in community-based activities with their peers, which decreases opportunities for their social learning and development. Parents of children with NDD seek out programs that address physical and psychosocial development. Several studies already support the positive effects for the child to attend physical activity programs (PAPs). However, these studies are highly prone to biases and Hawthorne effect. In the planning stage of a large prospective study to assess the effectiveness of PAPs we reviewed the records of children who participated in a gymnastic-based program, the Empowering Steps Movement Therapy (ESMT). Besides generating useful data for developing the prospective study we thought these data reflect the rate of changes in context of normal practice in a naturalistic environment; therefore protected from Hawthorne effect and other biases.

**Design:** This is a historical cohort: the files of 67 children with NDD were examined across a 2-year period (Jan 2011 to Jan 2013). As part of standard practice, the ESMT therapists document changes in motor function every 6 months, using the ESMT's proprietary motor scale. Parents also completed a parental questionnaire in June 2011 regarding their perceptions of changes in their child's physical and psychosocial function, as well as family functioning since their child started the program.

**Results:** Linear Mixed Effects Model clearly identified three groups according to changes in motor function: the ones with rapid changes (mostly functional children with autism spectrum disorder: *n* = 13), the ones with moderate changes (different types of NDD diagnoses: *n* = 41) and the ones that did not change or even decreased motor skills over the follow-up (children with complex diseases or uncontrolled epilepsy despite treatment: *n* = 13). Parental questionnaires (*n* = 39) reported improvement in most of the children's physical and psychosocial abilities; they also indicated improvement in some of the family parameters. There was no association between the changes in children's motor functions and parents' responses to the questionnaire.

**Conclusion:** Despite limitations due to the retrospective nature of the study, the absence of a control group and the absence of validated measurement tools, the observed positive effects of attending movement therapy center on motor performance and psychosocial development confirm in a naturalistic environment what has been shown in context of clinical trials or quasi-experimental studies. These results are not conclusive. They warrant further, rigorous investigation using validated instruments, independent assessors, and control groups.

## Introduction

The prevalence of neurodevelopmental disabilities (NDD) among children aged 4–14 years is ~3.6–12.8% depending on the definitions, and is increasing (1, 2). Morris et al. defines NDD as “*a group of congenital or acquired long-term conditions that are attributed to impairment of the brain and/or neuromuscular system and create functional limitations*” [p. 1105, (3)]. The impact of NDD on family and society is important due to ongoing functional needs, high level of medical attention, and challenges related to behavioral and emotional adjustments (4–8).

The primary disability and the presence of behavioral issues, which often accompany NDD, limit or even preclude children's participation in typical social and recreational activities with peers (8–10); therefore, they tend to miss these important aspects of social learning during the developmental period (11–13). Child isolation is a source of concern for their parents (14). Families often seek and enroll their children in programs aimed at stimulating both motor skills and psychosocial development, with the goal of improving social integration reaching their potential and improving their quality of life.

Adapted community-based physical activity programs (PAPs) for children with NDD offer the appropriate environment for enjoyable and meaningful inclusive recreational activities, thereby creating a context for socio-cultural learning and development (12). Literature on the effects of physical activity in children with NDD has shown a positive impact of physical activity on variety of outcomes such as cognitive function, social integration, friendship development and physical ability (15–22). However, the majority of these studies are limited because of the high risk of Hawthorn effect as well as observation and selection biases in context of trials or quasi-experimental studies. Other limitations include short follow-up and a narrow focus on limited range of outcomes. Further, they describe a large variety of PAP structures and strategies with different aims such as motor development (e.g. sport activity), recreation/leisure (e.g. bowling, dancing, and horseback riding) or both, which limits inferences. In context of preparing a prospective study we extracted data from one center that collects systematically the child's motor changes in a systematic way. These data offer a unique opportunity to assess motor changes in context of usual practice in a naturalistic environment and 2 years follow up, protected from observation biases and Hawthorne effects.

The Empowering Steps Movement Therapy (ESMT) program is a gymnastics-based motor intervention that operates in the Greater Vancouver Area, as a private organization serving ~125 children with NDD between 2 and 18 years of age, irrespective of diagnosis. The ESMT program employs a range of motor learning approaches, communication tools, and behavioral management techniques delivered by trained ESMT therapists. ESMT is an individualized, one to one, child-centered program that aims to enhance motor development, attachment and emotional state. Since its creation in 2002 as an offshoot of a large gymnastics center with a wide range of programs from early motor milestone programs to competitive gymnastics, ESMT has evolved and gained experience working with children with NDD. In January 2011, the program started to quantitatively measure each child's motor development every 6 months using a motor scale developed by VS. The ESMT motor scale (S1 Appendix) is comprised of eight progressive stages that are subdivided into 20 categories of movements that range from basic movements for non-ambulatory motor development, to more complex motor patterns and sequences designed to develop executive functioning. In 2011, based on the ESMT experience of observed changes, a developmental pediatrician (RW) developed a “Parental Questionnaire” to assess the parents' perception of ESMT on children and families. Parents completed this questionnaire in June 2011.

In 2015 our team at UBC British Columbia Children's Hospital Research Institute (BCCHRI) decided to conduct a prospective study of the children registered in the ESMT program with the objective of completing a rigorous review of the program's effects on children and parents. In the planning stage of this study, we decided to review the data collected on each child by RW and the ESMT staff during the period January 2011 to January 2013 to document the child's motor changes and the parents' perceived changes in child and family quality of life and functioning, collected in June 2011. We expected to observe improvement in complex motor functions, as well as in motivation, behavior, social integration, daily life functions, and quality of life, as perceived by parents. Finally, we anticipated a correlation between the improvement of motor functions and other psychological and social factors. However, our main objective was descriptive: to determine the reported magnitude of change, as well as identifying related factors, in preparation for the prospective study.

Despite the non-use of standard validated instruments to assess motor functions, we decided to publish these results because we feel they provide relevant information for the planning of other studies assessing the effects of community-based programs designed to improve daily function and quality of life of children with NDD and their families. Further, these data show the children's changes in context of usual practice over 2 years, therefore complementing the results from experimental studies, with the advantage of being protected from observation biases and Hawthorne effects. Finally, community-based PAPs are very unlikely to use standard motor assessment tools, because of the recurrent license fees that are not bearable for these organizations. This project was developed in collaboration with ESMT staff and RW, but the study design and data analysis were conducted in complete independence from the ESMT staff.

## Materials and Methods

### Participants

The ESMT files of 67 children with NDD (2–18 years of age) who attended the ESMT program between January 2011 (when systematic assessments of motor function began at ESMT) and January 2013 were considered for the study, regardless of diagnosis, as long as parents had signed the authorization for secondary use of data for research purpose. If a child started the ESMT training before Jan 2011, he/she was exposed to the program but no rigorous assessments were done prior to this date. The age range of the participants was 24–221 months (median = 81, mean = 89.9, *SD* = 43), and 59.7% of children were male. Twenty-six children (38.8%) had a sole diagnosis of Autism Spectrum Disorder (ASD) while 20 (29.8%) had ASD associated with other diagnoses, such as Attention Deficit Disorder, Seizure Disorder, or Down Syndrome. Eighteen children (26.9%) had other diagnoses such as Fetal Alcohol Spectrum Disorder (FASD), Cerebral Palsy (CP), or rare genetic disorders. The remaining three children (4.5%) had undiagnosed neurodevelopmental conditions. These diagnoses are based on the de-identified information provided by ESMT from their files without medical verification.

**Figure 1:**
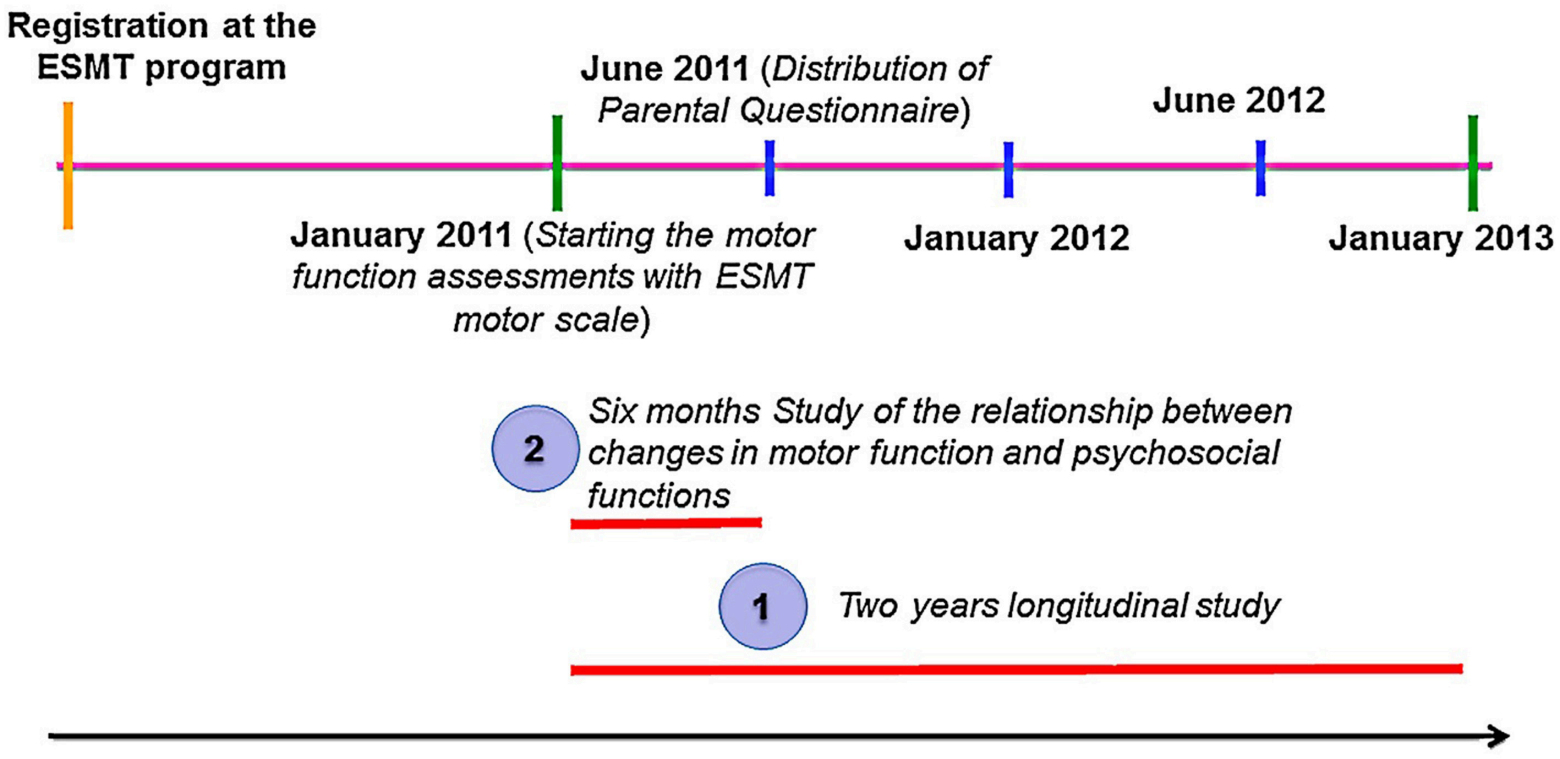
Study process. Part 1 refers to the study of changes in children's motor abilities from January 2011 to 2013 assessed by the ESMT™ (V1.0) motor scale. Part 2 is related to the six-month study (Jan 2011–June 2011) of children with NDD whose parents also completed the Parental Questionnaire in June 2011.

To assess the possible relationship between the changes in motor function and perceived changes in daily life function and quality of life (i.e., using the parental questionnaire), we selected the subgroup of children/families that were registered in the ESMT program in Jan/Feb 2011 and completed the Parental Questionnaire in June 2011 (*n* = 39). In this subgroup, we could study both the changes in motor function from Jan to June 2011 and the perceived changes in quality of life and daily function assessed in June 2011 (Figure 1).

### Procedures

The change in motor performance is based on the assessments made at 6-month intervals using the ESMT motor scale. The data were extracted from the chart by the ESMT staff. The researchers extracted information from the paper version of the anonymized completed parental questionnaires. The data analysis was conducted during the period of May-September 2015.

The study was reviewed and approved by the University of British Columbia/Children's and Women's Health Center of British Columbia Research Ethics Board. Since we received the de-identified data of children whose parents had signed authorization of use for research purposes, for our analysis, formal consent from families was not required under Canadian Tri-Council policy 2nd edition, article 5.5.

### ESMT Program Exposure

Most children with NDD received one training session per week ranging from 30 to 60 min. Absences were not recorded by the ESMT team at this time. Children's records showed that 18, 24, and 16% of participants had been involved in the program for 6–12, 12–24, and >24 months prior to their first ESMT motor assessments, respectively. The ESMT program is a personalized one to one intervention based on each child's specific needs and abilities as determined at their initial assessment prior to beginning intervention sessions. At the end of each session, the child's performance is recorded to guide the next session's training activity. A refined scaffolding process is in place, to reach optimal performance through continuous improvement ensuring that the child is always in their zone of proximal development. More details about the ESMT program can be found in Appendices S2,S3.

### Measures

Children's motor ability was measured using the ESMT® (V2.0) Motor Scale (hereafter referred to as the “ESMT scale”) every 6 months starting January 2011. The ESMT scale is a physical literacy roadmap for assessing motor ability with eight progressive stages. Each stage has 20 assessment skills for a total number of 160 skills (S1 Appendix), which allow the ESMT team to observe how children best develop motor skills and enhance their functional motor development. The ESMT scale starts at stage 1 (building motor milestones for non-ambulatory children) and progresses to Stage 8 (children are executing skills of a typical 12-year-old such as complex sequencing and the memorization of 10 skill routines, e.g., the trampoline routine is based on the compulsory routine for entry level competitive Trampoline athletes). Two ESMT staff completed the semi-annual assessments for each child.

The parental questionnaire was used to assess the parents' perceptions of changes in their child and in the family. Parents were asked to report on any perceived change since their child started the ESMT program on (i) their child's behavior and activity at home, changes in their ability to manage daily life activities, recreational inclusiveness, sleep patterns, mood, self-esteem and their quality of life; and (ii) family functioning and quality of life. Each item represented one domain. Responses were based on a 5-point Likert scale ranging from 1 “marked improvement” to 5 “significantly worse.”

The two instruments used for generating the motor function score and identifying changes in daily activities reflect the experience of the ESMT team, but neither the ESMT motor scale nor the parental questionnaire have been formally validated.

### Data Analysis

The analysis was completed using SPSS version 20. The 2011-2013 historical cohort enabled computing the motor function curve of each child over 2 years. Using Linear Mixed Effects Model with random effects for subject, we identified growth patterns and predictors. We also investigated the association between children's exposure to the ESMT training and average growth in motor function within 2 years of study, considering the effects of age at study entry, diagnosis, baseline ESMT score and duration of program attendance before the study.

We also assessed the association between the children's changes in motor function for the period Jan/Feb to June 2011 (expressed as percentage of change from baseline) and the parents' perceived changes in both the children's and parents' daily life functioning and quality of life in June 2011. We computed the frequency distribution of parents' responses to each question. To assess the possible association between the changes in motor function (ESMT scale score) and parents' perceived changes, we performed a Spearman's rank test.

## Results

**Figure 2:**
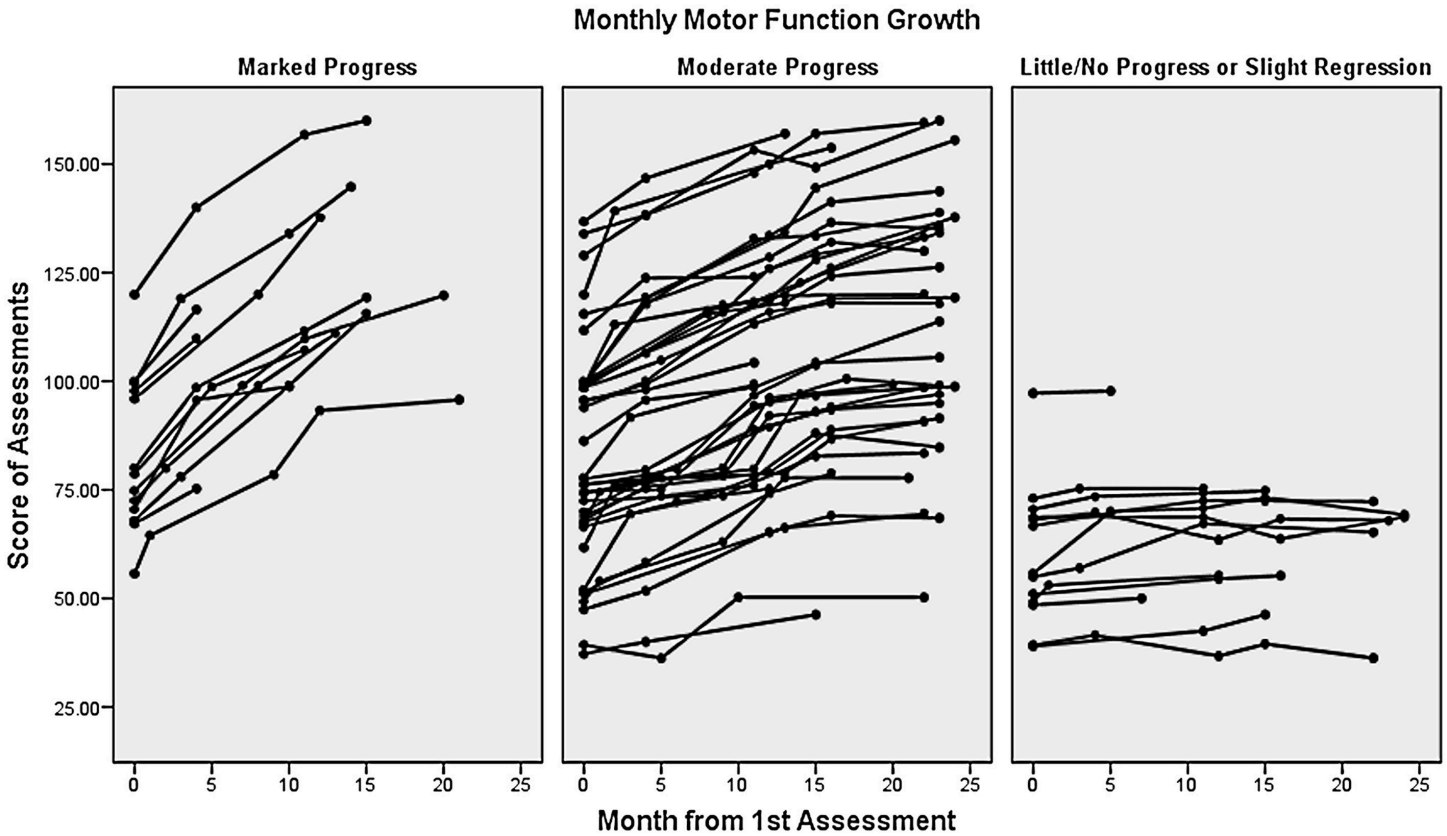
Motor function curves of 67 children from 2011 to 2013. The Y axis represents motor function scores measured by the ESMT scale; X axis is the number of months that children were exposed to the ESMT training after the first assessment. Three graphs represent the progress level of children.

In total, 70 children with NDD were registered at the ESMT program during the period Jan 2011 to Jan 2013. Three did not sign the ESMT program's research release form; hence, data from 67 children were used in the longitudinal analysis of the changes in children's motor function while attending the ESMT program. From those, 79% started their ESMT training before Jan/Feb 2011, 10.5% started in Jan/Feb 2011, and 10.5% after Jan/Feb 2011. Figure 2 shows the motor function trajectories of the 67 participants for the period Jan 2011 to Jan 2013. Out of 67 study participants, 36 children had a full 2 years of motor function assessments (i.e., 5 data points), 11 children had 4, 14 children had 3, and 6 children had 2 data points. For two children whose second motor function assessment score (June 2011) was missing, we used the last-value-carried-forward method, which assumed no change during the 6-month period (a conservative approach) to replace the missing data.

The Linear Mixed Effects Model shows the relationship between the ESMT program and motor function improvement, along with the respective effects of age at study entry, baseline score and duration of exposure to the ESMT program before the study: the monthly growth in ESMT motor function score is 1.30 (95% CI: 0.95–1.6); *p* < 0.0001. We categorized participants' progress level into three categories: the lowest 20th percentile (little/no progress or slight regression), the highest 20th percentile (marked improvement), and those in between (moderate progress). Neither age at study entry, baseline score or previous exposure to the program was associated with the change in motor function during the study (Table 1).

**Table 1:** Linear mixed effects model.

**Parameters**	**Estimate**	**Std. error**	**95% Confidence Interval**		***p*-value**
			**Lower bound**	**Upper bound**	
Intercept	1.93	57.91	−128.6	132.5	0.973
**ESMT program exposure**	**1.30**	**0.177**	**0.95**	**1.6**	**0.0001**
Baseline score at entry	1.00	0.926	−1.15	3.17	0.276
Previous attendance (months)	−0.014	1.72	−4.28	4.25	0.993
Age at study entry (months)	−0.0037	0.603	−1.5	1.50	0.995

*The bold values show that the monthly growth in ESMT motor function score is 1.30 score point with 95% CI: 0.95–1.6 and p < 0.0001*.

Linear Mixed Effects Model showing the effects of ESMT program exposure, age at study entry, previous attendance at the ESMT program and Baseline score. The Estimate describes the monthly change in ESMT scale score.

The Parental Questionnaires was completed by 39 parents (out of 67) in June 2011. The reasons 28 families did not complete the questionnaire include: lack of time or interest or absence from the program when the questionnaire was offered. There were no significant differences in the age and gender between children whose parents completed or did not complete the questionnaire. Completers were significantly more likely to have a sole diagnosis of ASD (56.4% for 39 compared to 17.8% for 28). Among 13 children who showed marked progress in motor function, 6 were in this subgroup of 39. Similarly, 2 children with no change and the one with a regression of motor functions were also part of this subgroup.

Parents reported improvement in most of the child's physical and psychosocial abilities (Table 2). They also indicated some improvement in few family parameters (Table 3). We did not find any association between the changes in children's motor functions and parents' responses to the questionnaire.

**Table 2:** Percentage of parents reported perception of changes in their child after attending the ESMT program (*n* = 39).

**Items**	**A**	**B**	**C**	**D**	**Items**	**A**	**B**	**C**	**D**
Fitness	47.2	44.4	8.3	–	Participation in school sports	42.9	23.8	33.3	–
Balance	60.5	31.6	7.9	–	Participation in school classroom activities	31.0	44.8	17.2	6.8
Coordination	54.1	40.5	5.4	–	Play skills	20	54.3	22.9	2.9
Muscle strength	48.6	42.9	8.6	–	Interest in other children	30.8	28.2	38.5	2.6
Anxiety	38.9	47.2	11.1	2.8					
Motivation	20.5	56.4	17.9	5.1	Forming friendships	22.9	31.4	40	5.7
Cooperation	25.6	53.8	15.4	5.1	Academic performance	32.1	39.3	25	3.6
Mood	23.1	43.6	25.6	7.7	Participation in community based sports	26.7	20	53	–
Self-confidence	39.4	45.5	12.1	3	Involvement in other recreational activities	20	44	36	–
Attention	28.9	52.6	15.8	2.6	Manual skills	21.2	48.5	30.3	–
Transitioning to a new activity	26.3	50	21.1	2.6	Sleep problems	10.3	24.1	51.7	13.7
Frequent or abnormal movements	6.9	34.5	51.7	3.4	Eating problems	8	44	48	–
Frequency of muscle spasm	0	50	50	–	Behavioral problems	20.6	38.2	38.2	2.9
Communication skills	27.8	52.8	19.4	–	Sibling Relationships	20.8	41.7	37.5	–
Participation in school physical education	36.0	44	15	4	Activities of daily living	21.1	44.7	31.6	2.6

*Column A: Percentage of parents that indicated “Marked improvement.” Column B: Percentage of parents that indicated “Some improvement.” Column C: Percentage of parents that indicated “About the same (no change).” Column D: Percentage of parents that indicated “Somewhat worse” and “Significantly worse.” “No response,” “I don't know,” and “Not applicable” were not valid responses and were not taken into consideration for the calculated percentages*.

**Table 3:** Percentage of parents reported perceptions of changes regarding their child and family after attending the ESMT program (*n* = 39).

**Items**	**A**	**B**	**C**	**D**
Your level of childcare stress	10.5	47.4	36.8	5.3
Level of concern about your child's health	14.3	34.3	45.7	5.7
Feelings about your child's quality of life	15.8	44.7	34.2	5.3
Feelings about your quality of life	14.7	35.3	41.2	8.8
Relationships within your household	17.1	34.3	42.9	5.8
Safety concerns about your child	7.9	47.4	42.1	2.6
Feelings about your child's future	15.4	35.9	35.9	12.8
Concerns about siblings	4.2	37.5	45.8	12.5

*Column A: Percentage of parents that indicated “Marked improvement.” Column B: Percentage of parents that indicated “Some improvement.” Column C: Percentage of parents that indicated “About the same (no change).” Column D: Percentage of parents that indicated “Somewhat worse” and “Significantly worse.” “No response,” “I don't know,” and “not applicable” were not valid responses and were not taken into consideration for the calculated percentages*.

## Discussion

This pilot observational feasibility study was conducted with a mixed group of children with NDD followed prospectively over 2 years while attending the ESMT program to assess changes in motor function and perceived changes in a wide range of outcomes in both children and families. Historical cohort design was possible because of the systematic semi-annual recording of motor performance by the ESMT therapists.

This feasibility study confirms the association observed in previous intervention studies (15–22) between participation in physical activity and improvement in motor performance among children with ASD (*n* = 46, with 20 having other conditions), as well as other NDD (18 with FASD, CP, or rare genetic disorders and 3 with undiagnosed conditions). The motor function curves (Figure 2) show consistent progress with large variations between children. The average monthly change in the motor function score during the 2-year study is independent of age at first assessment, baseline score and duration of previous exposure to the program. The 13 children with the most rapid change in motor functions (highest slope) had autism only. The children who regressed (*n* = 1) or did not improve (*n* = 2), had similar characteristics: they were young (between 2 and 7 years of age), non-cooperative during the sessions, and were complex cases, two with multiple NDD conditions and one with severe ASD combined with epilepsy. In the future, it will be important to determine whether the program can adequately address these more complex cases.

Parents report perceived benefits for children with NDD in a wide array of outcomes (Table 2).

These results however, need to be interpreted in light of numerous limitations, including the absence of a control group, possible selection biases, and the use of not validated measurements. The main reason for not using validated instruments in usual practice is budgetary, due to the scales' license fees. ESMT developed proprietary instruments in order to evaluate their children and assess the changes. We recognize the limitation of using an instrument that has not been properly evaluated, but we also recognize the unique data provided by this study because, to our knowledge, there is no community-based study of PAPs that followed all participants using costly standard tools. Further, many study results like the comparisons of different diagnosis categories; the absence of effects of previous ESMT attendance, or the minor role of the diagnosis compared to severity, are somewhat independent of the type of scale used. However, it is not the purpose of this article be conclusive with strong causal inference at this stage; our results rather confirm in natural context of normal practice the positive effect of attending PAPs for children with NDD. They also call for the conduct a large prospective study with appropriate control groups and validated measurement tools.

From the literature, several short-term studies have shown improved motor functions of children with CP attending different community programs (15, 16, 19, 20). In a randomized controlled trial (*n* = 99) Davis et al. showed the positive effect of a 10-week horseback riding on quality of life and physical functions (20). Another study showed the positive effect of a 6 months movement and swimming intervention on respiratory functions and water orientation skills of 46 children with CP (23). Similarly, a few studies showed the positive effects of horseback riding in children with autism using wait-time as control period (21, 22). One study with 42 children showed improvement in self-regulation, adaptive skills and motor skills over a 10-week period (21). Another study of 34 children showed improvements in their social function after 12 weeks, but the authors did not assess the changes in motor function (22). A small study (*n* = 3) examined the effects of a therapeutic skating intervention for children with ASD and showed improvement in physical functions (17). Finally, one study conducted in 90 young individuals (12–25 years of age) with physical disabilities showed the beneficial effects of a 6-months reverse-integrated basketball activity on quality of life and perceived social competence (24). These studies on children with different types of disabilities are interesting, but they generally focus on the changes of a limited number of outcomes in context of a specific intervention and do not assess the impact on families; the follow-up is generally not long (< 6 months), which increases the risk of a Hawthorne effect. Finally, in context of not blind randomized trial, observation bias is possible, besides Hawthorne effect.

Our study is more naturalistic and the historical design makes it less prone to observation biases: the ESMT therapists collected motor performance data for their internal use, 2 years before the study. The observed improvement in motor function over 2 years is certainly expected in context of an intervention which is one to one, personalized and based on each child's needs, with weekly evaluation to plan the next session for more efficient scaffolding (25, 26). It was interesting to also observe the parents' perceived positive change in a large range of psychosocial and daily function outcomes in both children (Table 2) and families (Table 3), at least in the subset that completed this measure. Conceptually, outcomes such as the ones reported in Table 2 are consistent with Activity Theory's social cultural model of learning and child development (27, 28). According to this theory, a child's learning happens in the context of social environment and community through a series of activities in which the child develops high executive functions such as self-regulation or working memory, and higher levels of social integration, communication, self-esteem, and daily life functions. The ESMT program offers an ideal, inclusive environment to foster the development of each child as a whole thereby creating a context for socio-cultural learning and development. As per the Transactional Model Theory (29) the child's improvement may have a positive impact on anxious and depressed parents, and subsequently the whole family system as evidenced in Table 3 (30). Improvement in the family's quality of life is an important outcome to consider in future studies. According to the World Health Organization's International Classification of Functioning, Disability and Health for Children and Youth (ICF-CY) (31, 32), family is the most important environmental factor for child development. (33).

The absence of association between changes in motor function over 6 months and parents' responses to the questionnaire likely reflects the complexity of child development with NDD, which is a dynamic, multifactorial and interactive process rather than fixed and predictable (34, 35). Therefore, it is difficult to associate the psychosocial changes to a single factor. The absence of an association may also reflect a lag time between changes in motor function and changes in psychological, functional or social outcomes that may take more than 6 months. Moreover, since the parental questionnaire was only conducted once, we could not assess the dynamic of changes and possible interactions between these factors. Lastly, although developed by field experts, we do not know the psychometric properties of the two instruments used to assess changes, which limits our understanding.

As already stated, the present study has important limitations. Four particular matters of concern are the absence of a control group, the possible selection bias, the absence of formal validation of the measurement tools and possible co-interventions that were not assessed. The absence of a control group limits the interpretation, as the observed changes in motor function are due in part to normal progression when aging. Consequently, we cannot precisely determine the program's effect. More useful than the average growth however, is the wide range of changes observed among children that may lead to the identification of children that may benefit most from the program, and a more accurate correspondence between specific program features and the type of NDD. Also, the fact that the motor change during the 2-year follow-up is independent of age and duration of program attendance is encouraging as it indicates that motor function may improve whatever the age, and does continue after several years in the program. Selection bias is another concern. Attending the ESMT program is voluntary for parents/families, which most likely self-select and pay to attend if they perceived the benefits. This possible prevalence bias however, did not remove heterogeneity in the study population as demonstrated by the large distribution of personal growth curve with no progression in a few children (Figure 2). Another self-selection bias may have occurred when 28 families did not complete the parental questionnaire, with participating parents being the ones most satisfied with the program, leading to a positive inflation of the results. However, there are also studies that show the reverse, with unsatisfied participants being the sub-group that self-selects. (36, 37) Overall, we believe this subgroup of 39 families (56%) is a fair representation of the population that attended the program in 2011.

Furthermore, the evaluation tools used by the ESMT program have not been formally evaluated for their psychometric properties. This limitation raises questions regarding the meaning of a change in motor functions. Also, it does not authorize a valid comparison with other studies that used standard tools like BOT-2 for instance. We already stressed the fact that the use of validated tools by community sites in usual practice is not a real option for most sites because of the recurrent license's fees. It is for this specific reason that ESMT developed its own proprietary scale as a practical tool to evaluate the children's progress and guide the intervention. The ESMT motor scale, has been developed by experienced professionals with a solid background in child development, kinesiology and gymnastics, to assess motor performance changes and guide practice, which ensures high content validity. In addition, the same motor scale was used from Jan 2011 to Jan 2013, therefore ensuring consistency in the assessments. This contextual information suggests that a scoring bias is not likely. Finally, many useful results are independent of the scale used such as the poor progress of children with complex disease, the relative limited importance of diagnosis category compared to disease severity to explain the change, or the continuous change whatever the duration of previous ESMT attendance, which is encouraging.

The parental questionnaire developed by RW, an expert in Pediatric Medicine, also bears high content validity; the questions were focused and clear with simple wording to prevent misinterpretation. RW used the method we use when we adapt a “satisfaction survey” to a new population. The specific wording is changed to reflect the new context, but the validity of the modified instrument is not questioned.

Finally, Because of the retrospective nature of this study, we have not been able to collect information regarding the other concurrent activities of children. Without having precise assessment of possible co-interventions, interpreting the meaning and significance of observed changes is challenging.

## Conclusion

Results from this feasibility pilot observational study cannot be taken as solid evidence of a causal relationship between the ESMT training and changes in children/youth's and families' function and quality of life. The main limitation is the use of measurement instruments that had not been validated before. Therefore, study results should be considered with precaution, because we do not know whether they reflect a real change (i.e., a true ESMT outcome) or they are still in the area of the measurement errors of the ESMT scale. Despite this major limitation the study shows that over 2 years of practice children with different diagnosis progress at different pace and few of them regress. It shows the importance of disease severity in limiting motor skills improvement and it shows that improvement is independent of previous attendance of the program. This study is aimed at stressing the importance of considering physical activity for children with NDD and to promote research in this area. Further studies might consider assessing different sub-groups defined by diagnosis, severity, or age, for instance. Besides assessing changes in motor abilities, we also suggest assessing psychosocial and mental outcomes in both the child and the family.

## Ethics Statement

This study was carried out in accordance with the recommendations of the Canadian Tri-Council Policy. The protocol was approved by the University of British Columbia/Children's and Women's Health Center Research Ethics Board.

Since we worked with data extracted from patients' files and data were anonymized, there was no need to obtain consent from families (article 5.5 of the 2nd edition of the Tri-Council Policy Statement: Ethical Conduct for Research Involving Humans of Canada).

## Author Contributions

MG and J-PC took a leadership role in developing proposal and getting funding as well as managing the project. MG conducted the statistical analysis. J-PC reviewed results and commented. Both MG and J-PC took Leadership in developing the article. WM, AMi, JW, AMa, LM, and RW reviewed protocol for grant submission, reviewed analyses results, and actively participated in reviewing and commenting the article. RW led the initiative as community pediatrician. RB developed the analysis section of the protocol and supervised the statistical analysis. VS facilitated the research organization at Club Aviva.

### Conflict of Interest Statement

VS is the director and founder of the Empowering Steps Movement Therapy (ESMT) program owned by Symington Teaching Programs and delivered at Club Aviva Recreation Ltd. where the study was conducted. VS is involved as a co-author for her specific expertise but did not play any decisional role regarding the publication. The remaining authors declare that the research was conducted in the absence of any commercial or financial relationships that could be construed as a potential conflict of interest.

## Supplementary Material

S1 Appendix Introduction to the Empowering Steps Movement Therapy® (V2.0) Motor Scale.

S2 Appendix ESMT scientific protocol: visual representation.

S3 Appendix Overview of the ESMT program.

## References

[1] Halfon N Houtrow A Larson K Newacheck PW. The changing landscape of disability in childhood. Future Child. (2012) 22:13–42. 10.1353/foc.2012.0004 22550684

[2] Morris C Simkiss D Busk M Morris M Allard A Denness J. Setting research priorities to improve the health of children and young people with neurodisability: a British Academy of Childhood Disability-James Lind Alliance Research Priority Setting Partnership. BMJ Open. (2015) 5:e006233. 10.1136/bmjopen-2014-006233 25631309 PMC4316435

[3] Morris C Janssens A Tomlinson R Williams J Logan S. Towards a definition of neurodisability: a Delphi survey. Dev Med Child Neurol. (2013) 55:1103–8. 10.1111/dmcn.12218 23909744

[4] Anderson D Dumont S Jacobs P Azzaria L. The personal costs of caring for a child with disability: a review of the literature. Public Health Rep. (2007) 122:3–16. 10.1177/003335490712200102 17236603 PMC1802121

[5] Brehaut JC Kohen DE Garner RE Miller AR Lach LM. Health among caregivers of children with health problems : findings from a canadian population-based study. Am J Public Health. (2009) 99:1254–63. 10.2105/AJPH.2007.129817 19059861 PMC2696656

[6] Stabile M Allin S. The economic costs of childhood disability. Future Child. (2012) 22:65–96. 10.1353/foc.2012.0008 22550686

[7] Ginsburg KR. The importance of play in promoting healthy child development and maintaining strong parent-child bonds. Pediatrics. (2007) 119:182–91. 10.1542/peds.2006-2697 17200287

[8] Statistics C Participation and Activity Limitation Survey 2006: families of children with disabilities in Canada. Stat Canada Cat. (2008). 6–20.

[9] Majnemer A Shevell M Law M Birnbaum R Chilingaryan G Rosenbaum P. Participation and enjoyment of leisure activities in school-aged children with cerebral palsy. Dev Med Child Neurol. (2008) 50:751–8. 10.1111/j.1469-8749.2008.03068.x 18834388

[10] Kang L-J Palisano RJ King G a Chiarello LA. A multidimensional model of optimal participation of children with physical disabilities. Disabil Rehabil. (2014) 36:1735–41. 10.3109/09638288.2013.863392 24325580

[11] Leach J Scott P Individual and Sociocultural Views of Learning in Science Education. Sci Educ. (2003) 12:91–113. 10.1023/A:1022665519862

[12] John-Steiner V Holbrook M Sociocultural approaches to learning and development_ A Vygotskian framework. Educ Psychol. (1996) 31:191–206.

[13] Sun J How object, situation and personality shape human attitude in learning: An activity perspective and a multilevel modeling approach. Learn Individ Differ. (2009) 19:314–9. 10.1016/j.lindif.2009.02.002

[14] Davey H Imms C Fossey E “Our child's significant disability shapes our lives”: experiences of family social participation. Disabil Rehabil. (2015) 8288:1–8. 10.3109/09638288.2015.1019013 25738914

[15] Casady RL Nichols-Larsen DS. The effect of hippotherapy on ten children with cerebral palsy. Pediatr Phys Ther. (2004) 16:165–72. 10.1097/01.PEP.0000136003.15233.0C 17057544

[16] Cook O Frost G Twose D Wallman L Falk B Galea V. CAN-flip: a pilot gymnastics program for children with cerebral palsy. Adapt Phys Avtivity Q. (2005) 32:349–70. 10.1123/APAQ.2015-0026 26485738

[17] Casey AF Quenneville-Himbeault G Normore A Davis H Martell SG. A therapeutic skating intervention for children with autism spectrum disorder. Pediatr Phys Ther. (2015) 27:170–7. 10.1097/PEP.0000000000000139 25822357

[18] Garcia-Gomez A Lopez Risco M Rubio JC Guerrero E Garcia-Pena IM Effects of a program of adapted therapeutic horse-riding in a group of autism spectrum disorder children. Electron J Res Educ Psychol. (2014) 12:107–28. 10.14204/ejrep.32.13115

[19] McGibbon NH Benda W Duncan BR Silkwood-Sherer D. Immediate and long-term effects of hippotherapy on symmetry of adductor muscle activity and functional ability in children with spastic cerebral palsy. Arch Phys Med Rehabil. (2009) 90:966–74. 10.1016/j.apmr.2009.01.011 19480872

[20] Davis E Davies B Wolfe R Raadsveld R Heine B Thomason P. A randomized controlled trial of the impact of therapeutic horse riding on the quality of life, health, and function of children with cerebral palsy. Dev Med Child Neurol. (2009) 51:111–9. 10.1111/j.1469-8749.2008.03245.x 19191844

[21] Gabriels RL Agnew JA Holt KD Shoffner A Zhaoxing P Ruzzano S Pilot study measuring the effects of therapeutic horseback riding on school-age children and adolescents with autism spectrum disorders. Res Autism Spectr Disord. (2012) 6:578–88. 10.1016/j.rasd.2011.09.007

[22] Bass MM Duchowny CA Liabre MM. The effect of therapeutic horseback riding on social functioning in children with autism. J Autism Dev Disord. (2009) 39:1261–90. 10.1007/s10803-009-0734-3 19350376

[23] Hutzler Y Chacham A Bergman U Szeinberg A. Effects of a movement and swimming program on vital capacity and water orientation skills of children with cerebral palsy. Dev Med Child Neurol. (1998) 40:176–81. 9566654 10.1111/j.1469-8749.1998.tb15443.x

[24] Hutzler Y Chacham-Guber A Reiter S. Psychosocial effects of reverse-integrated basketball activity compared to separate and no physical activity in young people with physical disability. Res Dev Disabil. (2013) 34:579–87. 10.1016/j.ridd.2012.09.010 23123871

[25] Stone CA. The metaphor of scaffolding : its utility for the field of learning disabilities. J Learn Disabil. (1998) 31:344–64. 9666611 10.1177/002221949803100404

[26] Verenikina I Understanding Scaffolding and the ZPD in Educational Research. Proceedings of the International Education Research Conference (AARE - NZARE) Auckland (2003).

[27] Devane B Squire KD Activity Theory in the Learning Technologies. In: Jonassen D and Land S, editors. Theoretical Foundations of Learning Environments, 2nd ed. New York, NY: Routledge (2012). p. 242–67.

[28] Nardi BA Studying context: a comparison of activity theory, situated action models, and distributed cognition. In: Context and Consciousness: Activity Theory and Human-Computer Interaction. Cambridge, MA: MIT Press (1996). p. 69–102.

[29] Fruzzetti AE Shenk C Hoffman PD. Family interaction and the development of borderline personality disorder : a transactional model. Dev Psychopathol. (2005) 1007–30. 10.10170/S0954579405050479 16613428

[30] Patterson JM Families experiencing stress. Fam Syst Med. (1988) 6:202–37.

[31] Rosenbaum P Gorter JW. The “F-words” in childhood disability: I swear this is how we should think! Child Care Health Dev. (2012) 38:457–63. 10.1111/j.1365-2214.2011.01338.x 22040377

[32] Cerniauskaite M Quintas R Boldt C Raggi A Cieza A Bickenbach JE. Systematic literature review on ICF from 2001 to 2009: its use, implementation and operationalisation. Disabil Rehabil. (2011) 33:281–309. 10.3109/09638288.2010.529235 21073361

[33] Rosenbaurn P King S Law M King G Evans J Family-centred service: a conceptual framework and research review. Haworth Press Inc. (1998) 18:1–20.

[34] Davies D Child Development: A Practitioner's Guide. 3rd ed. In: Webb NB, editor. New York, NY: The Guilford Press (2011). p. 3–130.

[35] van Geert P The contribution of complex dynamic systems to development. Child Dev Perspect. (2011) 5:273–8. 10.1111/j.1750-8606.2011.00197.x

[36] Bornehag C-G Sundell J Sigsgaard T Janson S. Potential self-selection bias in a nested case-control study on indoor environmental factors and their association with asthma and allergic symptoms among pre-school children. Scand J Public Health. (2006) 34:534–43. 10.1080/14034940600607467 16990165

[37] Aschengrau A Seage G Bias. Essentials of Epidemiology in Public Health. 3rd ed. Burlington, VT: Jones and Bartlett Learning (2013). p. 265.

