# Supplementary material for: Gymnastic-Based Movement Therapy for Children With Neurodevelopmental Disabilities: Results From a Pilot Feasibility Study
Source: Front Pediatr. 2019 May 14;7:186. doi: 10.3389/fped.2019.00186 (PMC6527593; doi:10.3389/fped.2019.00186)
Supplement: S1 Appendix — Introduction to the Empowering Steps Movement Therapy® (V2.0) Motor Scale. [file Data_Sheet_1.PDF]

EMPOWERING STEPS®

© Symington Teaching Programs Inc. 2008

Introduction to the Empowering Steps Movement Therapy® (V2.0) Motor Scale

The ESMT® (V2.0) motor scale (hereafter referred to as the ESMT scale) is designed as a physical literacy roadmap for developing motor ability with eight progressive stages. Each stage has 20 assessment skills for a total number of 160 assessment skills, which when assessed, allow us to observe how children best develop motor skills and how to facilitate functional motor development. The ESMT scale starts at stage 1 building motor milestones for non-ambulatory children and scaffolds learning through to Stage 8 where children are executing skills of a typical 12 year old such as complex sequencing and the memorization of 10 skill routines (the trampoline routine is based on the compulsory routine for entry level competitive Trampoline athletes).

The ESMT motor scale was designed to be very sensitive to measuring changes in motor functioning over time. The depth of skills and stages allows this program to be useful in motor development for all children with neuro-developmental disabilities regardless of their diagnosis. The ESMT scale was designed to provide a non categorical approach for the treatment for children and youth because it assesses motor development by function rather than by diagnosis.

1	Apparatus	Score
**Category 1: Forward rocking on the roller**: not at all = 0.0, to touch hands to the floor = .25, to allow the feet to come off of the floor = .50, to allow both hands and feet to come off of the floor = .75, supporting weight on hands and feet = 1.0	Lying and Rolling	
**Category 2: Sideward rocking on the roller alternating touching left and right foot while maintaining balance:** not at all =0.0, 2 person assist =.25, 1 person assist =.50,light prompt = .75, unassisted =1.0	Lying and Rolling	
**Category 3: Backward rocking on the roller:** not at all = 0.0, to touch hands or lift feet from the floor = .25, to allow hands and feet to come off of the floor = .50, to support weight on hands and feet = .75, support weight and push off from hands and feet = 1.0	Lying and Rolling	
**Category 4: In Prone position: lifts head upright, in Supine position turns head L and R with extremities symmetrical, in Supine position lifts head 45°:** not at all = 0.0, 2 person assist = .25, 1 person assist = .50,light prompt = .75, unassisted =1.0	Lying and Rolling	
**Category 5: In supine position brings hands to midline, fingers to each other**: not at all = 0.0, 2 person assist = .25, 1 person assist = .50,light prompt = .75, unassisted =1.0	Lying and Rolling	
**Category 6: In supine flexes R hip and knee through full range, repeats with the Left hip and knee:**not at all = 0.0, 2 person assist =.25, 1 person assist = .50,light prompt = .75, unassisted =1.0	Lying and Rolling	
**Category 7: Supine position: Reaches out with R arm and crosses the midline to reach for a toy, repeats with the L arm:** not at all = 0.0, 2 person assist = .25, 1 person assist = .50,light prompt = .75, unassisted =1.0	Lying and Rolling	
**Category 8: Supine position: Rolls over the right side, Roll over the left side:** not at all = 0.0, 2 person assist = .25, 1 person assist = .50,light prompt = .75, unassisted =1.0	Lying and Rolling	
**Category 9: Prone position: Rolls over the right side, Rolls over the left side:** not at all = 0.0, 2 person assist = .25, 1 person assist =.50,light prompt =.75, unassisted =1.0	Lying and Rolling	
**Category 10: Prone position: On forearms: lift head upright, extend elbows and lift and raise torso, on forearms weight on R, fully extend the left arm, repeat with L arm fully extended:** not at all = 0.0, 2 person assist = .25, 1 person assist = .50,light prompt =.75, unassisted =1.0	Lying and Rolling	
**Category 11: Supine position: Hands grasped by therapist: pulls to a sitting position with head controlled:** not at all = 0.0, 2 person assist = .25, 1 person assist = .50,light prompt = .75, unassisted =1.0	Sitting	
**Category 12: Supine position: Rolls to right side and attains sitting position, repeat to left side:** not at all =0.0, 2 person assist = .25, 1 person assist =.50,light prompt = .75, unassisted =1.0	Sitting	

1	Apparatus	Score
**Category 13: Sit on a mat supported by the therapist: lift head upright and maintain balance for 10sec: 0-2 sec = 0.0, 3-4 sec. = .25, 5-6 sec =.50, 7-8 sec. = .75, 9-10 sec =1.0**	Sitting	
**Category 14: Sit on a mat with arms propping for 10 sec.: 0-2 sec =0.0, 3-4 sec. = .25, 5-6 sec = .50,7-8 sec. = .75, 9-10 sec =1.0**	Sitting	
**Category 15: Sit on a mat: lower body to prone with control:** not at all =0.0, 2 person assist = .25, 1 person assist =.50,light prompt = .75, unassisted =1.0	Sitting	
**Category 16:Prone position: creep forward 6 ":** not at all = 0.0, 2 person assist = .25, 1 person assist = .50, light prompt = .75, unassisted =1.0	Crawling and Kneeling	
**Category 17: Doggy position: Maintain weight on hands and feet for 10 sec:** not at all = 0.0, 2 person assist =.25, 1 person assist = .50,light prompt = .75, unassisted =1.0	Crawling and Kneeling	
**Category 18: Doggy position: Attains sit arms free:** not at all = 0.0, 2 person assist = .25, 1 person assist = .50,light prompt = .75, unassisted =1.0	Crawling and Kneeling	
**Category 19: Prone position: Attains a doggy position with weight on both hands and feet:** not at all = 0.0, 2 person assist = .25, 1 person assist = .50,light prompt =.75, unassisted =1.0	Crawling and Kneeling	
**Category 20: On the floor: pulls to stand at a bench:** not at all = 0.0, 2 person assist =.25, 1 person assist =.50,light prompt = .75, unassisted =1.0	Walking and Jumping	

2	Apparatus	Score
**Category 1: Wheelbarrow walk forwards on the roller 5 steps:** no = 0.0, 2 steps = 0.25, 3 steps = 0.50, 4 steps = 0.75, 5 steps = 1.0	lying and rolling	
**Category 2: Prone position: Pivots Right 90°, Pivots Left 90°:** not at all = 0.0, 2 person assist = 0.25, 1 person assist = 0.50,light prompt = 0.75, unassisted = 1.0	lying and rolling	
**Category 3: On a mat, sits hands free for 10 sec.:** not at all = 0.0, 2 person assist = 0.25, 1 person assist = 0.50,light prompt = 0.75, unassisted = 1.0	Sitting	
**Category 4: Sitting hands free: Pick up a toy in front of the body to the R at 45°, to the L at 45° and behind the body to the R at 45°, to the L at 45°: R at 45°, to the L at 45°:** not at all = 0.0, 2 person assist = 0.25, 1 person assist = 0.50,light prompt = 0.75, unassisted = 1.0	Sitting	
**Category 5: Hands free execute a right side sit for 5 sec. and a left side sit for 5 sec.**: 1=0.0, 2=.25, 3=.50, 4=.75, 5= 1.0	Sitting	

**Category 6: From a sitting position rollover to attain a doggy position to the R and the L:** not at all = 0.0, 2 person assist = 0.25, 1 person assist = 0.50,light prompt = 0.75, unassisted = 1.0	Sitting	
**Category 7: Sit on a bench arms free for 10 sec.:** 0-2= 0.0, 3-4= .25, 5-6= .50, 7-8 = .75, 9-10 = 1.0	Sitting	
**Category 8: Trampoline: maintain seated bounce for 10 bounces, maintain doggy bounce for 10 bounces:** 0-2= 0.0, 3-4= 0.25, 5-6= .50, 7-8 = .75, 9-10 = 1.0	Sitting	
**Category 9: From a doggy position reach forward with the R arm above shoulder level, repeat with the L:** not at all = 0.0, 2 person assist = 0.25, 1 person assist = 0.50,light prompt = 0.75, unassisted = 1.0	Crawling and Kneeling	
**Category 10: In a doggy position: Crawl forward 6":** not at all: 0.0, assisted: .25, prompted: .50, hitch step unassisted: .75, reciprocal arms: 1.0	Crawling and Kneeling	
**Category 11: Crawl up 4 steps and crawl backwards down 4 steps:** not at all = 0.0, 2 person assist = 0.25, 1 person assist = 0.50,light prompt = 0.75, unassisted = 1.0	Crawling and Kneeling	
**Category 12: From a sitting position attain a high knee using arms and maintain balance for 10 sec.:** 0-2= 0.0, 3-4= .25, 5-6= .50, 7-8 = .75, 9-10 = 1.0	Crawling and Kneeling	
**Category 13: High knee position: attain 1/2 knee on the R using arms at maintain balance for 10 sec., repeat on the L:** 0-2= 0.0, 3-4= .25, 5-6= .50, 7-8 = .75, 9-10 = 1.0	Crawling and Kneeling	
**2**	**Apparatus**	**Score**
**Category 14: Standing: Maintain balance for 20 sec. hands free;** 0-4= 0.0, 5-9= .25, 10-14= .50, 15-19 = .75, 20+ = 1.0	Standing	
**Category 15: In a standing position holding onto a bench with 1 hand, lift the R foot and hold 5 sec, repeat on the L:** 1=0.0, 2=.25, 3=.50, 4=.75, 5= 1.0	Standing	
**Category 16: Standing holding onto a block with 2 hands, cruise 5 steps to the Left, repeat to the Right:** 1=0.0, 2=.25, 3=.50, 4=.75, 5= 1.0	Walking and Jumping	
**Category 17: Walk forward 10 steps:** not at all = 0.0, 2 person assist = 0.25, 1 person assist = 0.50,light prompt = 0.75, unassisted = 1.0	Walking and Jumping	
**Category 18: Trampoline: Leaning on a roller activate jumping 10x:** 0-2= 0.0, 3-4= .25, 5-6= .50, 7-8 = .75, 9-10 = 1.0	Walking and Jumping	
**Category 19: Trapeze: Support full weight in a hang:** not at all= 0.0, 2 person assist= .25, holding 2 hands= .50, holding 1 hand = .75, unassisted = 1.0	Trapeze	
**Category 20: Wall Bar: Climb up and down 3 steps of the wall bar:** not at all = 0.0, 2 person assist = 0.25, 1 person assist = 0.50,light prompt = 0.75, unassisted = 1.0	Wall Bar	

3	Apparatus	Score
**Category 1: 10x drumstick sit ups unassisted:** 0-2 = 0, 3-4 = 0.25, 5-6 = 0.50, 7-8 = 0.75, 9-10 = 1.0	lying and rolling	
**Category 2: Log roll down an incline left and right :** no = 0.0, 2A = 0.25, 1A = 0.50, light prompt = 0.75, unassisted = 1.0	lying and rolling	
**Category 3: From a stand attain a sit on a block:** no = 0.0, 2A = 0.25, 1A = 0.50, light prompt = 0.75, unassisted = 1.0	sitting	
**Category 4: From the floor, attain a sitting position on a large block:** no = 0.0, 1 person assist = 0.25, light prompt= .50, small block unassisted = 0.75, large block unassisted = 1.0	sitting	
**Category 5: From a high knee position walk forward 10 steps:** 0-2x = 0.0, 3-4x = 0.25, 5-6x = 0.50, 7-8x = 0.75, 9-10x = 1.0	Crawling and kneeling	
**Category 6: Beam 12" wide: Crawl forward 5 feet unassisted:** 0-1= 0.0, 2 = 0.25, 3 = 0.50, 4 = 0.75, 5 = 1.0	Crawling and kneeling	
**Category 7: Crawling up and down an inclined 12 inch beam for 8 feet (at a 20 cm height):** no = 0.0, 2A = 0.25, 1A = 0.50, light prompt = 0.75, unassisted = 1.0	Crawling and kneeling	
**Category 8: Trampoline: Maintaining a doggy position on the trampoline for at least 10 bounces:** no = 0.0, 2A = 0.25, 1A = 0.50, light prompt = 0.75, unassisted = 1.0	Crawling and kneeling	
**Category 9: From a hands free stand stoops down and picks up a toy from the floor:** no = 0.0, 2A = 0.25, 1A = 0.50, light prompt = 0.75, unassisted = 1.0	standing	
**Category 10: From a stand lower to a sit on the floor arms free, from a stand lower to a squat position arms free:** no = 0.0, 2A = 0.25, 1A = 0.50, light prompt = 0.75, unassisted = 1.0	standing	
**Category 11: From a high knee position, attain a stand through 1/2 knee position hands free, Left and right:** no = 0.0, 2A = 0.25, 1A = 0.50, light prompt = 0.75, unassisted = 1.0	standing	
**Category 12: From a sit on a block, attain a stand arms free:** no = 0.0, 2A = 0.25, 1A = 0.50, light prompt = 0.75, unassisted = 1.0	standing	
**Category 13: In a standing position, lift L foot and hold 10 sec., repeat on R:** 0-2x = 0.0, 3-4x = 0.25, 5-6x = 0.50, 7-8x = 0.75, 9-10x = 1.0	standing	
**Category 14: From a stand, step over a stick at knee height with R and with L:** no = 0.0, 2A = 0.25, 1A = 0.50, light prompt = 0.75, unassisted = 1.0	walking and jumping	
**Category 15: From a stand, kick a ball with the R foot and with the L foot:** no = 0.0, 2A = 0.25, 1A = 0.50, light prompt = 0.75, unassisted = 1.0	walking and jumping	
**Category 16: Stairs: Holding onto a railing: take 4 steps up and 4 steps down with alternating feet:** no = 0.0, 2A = .25, 1A =.50, light prompt = .75, unassisted =1.0	walking and jumping	

3	Apparatus	Score
**Category 17: Walk forward on a line 3/4" thick for 10 feet:** 0-2x = 0.0, 3-4x = 0.25, 5-6x = 0.50, 7-8x = 0.75, 9-10x = 1.0	walking and jumping	
**Category 18: Walk forward 10 feet between 2 parallel lines 8" apart:** 0-2x = 0.0, 3-4x = 0.25, 5-6x = 0.50, 7-8x = 0.75, 9-10x = 1.0	walking and jumping	
**Category 19: Walk sideways 5 feet left, repeat 5 feet right:** 1=0.0, 2=.25, 3=.50, 4=.75, 5= 1.0	walking and jumping	
**Category 20: Trampoline: Holding onto a hoop jump 20x:** 0-4x = 0.0, 5-9x = 0.25, 10-14x = 0.50, 15-19x = 0.75, 20+ = 1.0	walking and jumping	

4	Apparatus	Score
**Category 1: Backwards roll off a mat edge to land on knees:** no = 0.0, 2A = .25, 1A = .50, light prompt = .75, unassisted = 1.0	mushroom	
**Category 5: Balance on an upside down mushroom in a doggy position(Side to Side, Front to Back, Spin)**: no = 0.0, 2A = .25, 1A = .50, light prompt = .75, unassisted = 1.0	mushroom	
**Category 2: Modified plank on raised surface body perpendicular to hands on floor, upper thighs on the raised surface and hold for 10 sec (body parallel to the floor, arms perpendicular to form a 90° angle).**: 0-2 sec. = 0.0, 3-4 sec. = .25, 5-6 sec. = .50, 7-8 sec. = .75, 9-10 sec. = 1.0	roller	
**Category 3: On the roller, wheelbarrow walk forward 6 steps and then backwards 6 steps:** less than 6 steps = 0.0, 6 steps forward = .25, 6 steps forward and 2 backwards = .50, 6 steps forward and 4 steps backwards = .75, 6 steps forward and 6 steps backwards = 1.0	roller	
**Category 4: Crawl forward on a 6 inch beam elevated 20 cm (8") 16 feet:** no = 0.0, 2A = .25, 1A = .50, light prompt = .75, unassisted = 1.0	6" beam	
**Category 6: Walk up and down an inclined 6 inch beam for 8 feet (at a 20 cm.(8") height):** no = 0.0, 2A = .25, 1A = .50, light prompt = .75, unassisted = 1.0	6" beam	
**Category 7: Walk on a 6 inch beam which elevated at 80 cm off of the floor for 16 feet:** no = 0.0, 2A = .25, 1A = .50, light prompt = .75, unassisted = 1.0	6" beam	
**Category 9: Jumps from 2 feet over a rope on the air track:** no = 0.0, 2A = .25, 1A = .50, light prompt = .75, unassisted = 1.0	air track	
**Category 16: Climbs the dowel ladder on the airtrack:** no = 0.0, 2A = .25, 1A = .50, light prompt = .75, unassisted = 1.0	air track	
**Category 10: Jumps off a low box from 2 feet to land on 2 feet:** no = 0.0, 2A = .25, 1A = .50, light prompt = .75, unassisted = 1.0	block	

**Category 20: From a stand on the incline reach down and execute a forward roll:** no = 0.0, 2A = .25, 1A = .50, light prompt = .75, unassisted = 1.0	incline mat	
**Category 8: Descending the stairs using alternating feet:** no = 0.0, 2A = .25, 1A = .50, light prompt = .75, unassisted = 1.0	stairs	
**Category 11: Jumping on the trampoline from 2 feet to 2 feet for more than 20 consecutive bounces:** 5 or less = 0.0, 6-9 = .25, 10-14 = .50, 15-20 = .75, 21 or more = 1.0	trampoline	

4	Apparatus	Score
**Category 12: Jumps in a circle clockwise and counterclockwise on the trampoline:** no = 0.0, 2A = .25, 1A = .50, light prompt = .75, unassisted = 1.0	trampoline	
**Category 13: Initiating eye contact during partner bouncing on the trampoline to a song:** will not participate = 0.0, will participate but makes no eye contact = .25, brief eye contact = .50, sustained eye contact = .75, sustained eye contact & gesture or sound = 1.0	trampoline	
**Category 14: Copying 2 of: tuck jump,star jump position or 2 different sequence of arm actions on the trampoline:** No = 0.0, 1 occasionally = .25, 1 consistently = .5, 2 occassionally = .75, 2 consistently = 1.0	trampoline	
**Category 15: Jumps forwards along midline on the trampoline:** no = 0.0, 2A = .25, 1A = .50, light prompt = .75, unassisted = 1.0	trampoline	
**Category 17: Climbs up the wall bar 5 rungs and down the wall bar 5 rungs:** 0-2 rungs = 0.0, 3-4 rungs = .25, 5-6rungs = .50, 7-8 rungs = .75, 9-10 rung s= 1.0	wall bar	
**Category 18: Swings from an elevated box out and back holding on to a trapeze:** no = 0.0, 2A = .25, 1A = .50, light prompt = .75, unassisted = 1.0	trapeze	
**Category 19: Swings from an elevated box to land on 2 feet on a landing surface:** no = 0.0, 1A or 2A = .25, light prompt = .50, unassisted but not balanced = .75, unassisted to stable landing: 1.0	trapeze	

5	Apparatus	Score
**Category 1: Modified plank on a 20cm (8") height, hands on the floor, toes on the block, shoulder angle at 90° , torso flat and hold for 10 sec:** 0-2 sec = 0.0, 3-4 sec = 0.25, 5-6 sec = .50, 7-8 sec = .75, 9-10 sec = 1.0	roller	
**Category 6: Balance on an upside down mushroom while standing for 10 sec:** no = 0.0, 2A = .25, 1A = .50, light prompt = .75, unassisted = 1.0	mushroom	
**Category 2: Bear walk forward, backwards and sideways (L&R) on low parallel bars:** no = 0.0, 2A = .25, 1A = .50, light prompt = .75, unassisted = 1.0	P bars	

**Category 9: Log roll down an incline mat L&R with arms and legs extended squeezing a foam between hands:** no = 0.0, 2A = .25, 1A = .50, light prompt = .75, unassisted = 1.0	Incline mat	
**Category 3: Crawl 16 feet on a 6 inch beam raised 80cm backwards:** no = 0.0, 2A = .25, 1A = .50, light prompt = .75, unassisted = 1.0	6" beam	
**Category 4: Crawl 16 feet on a 6 inch beam sidewards (L&R) raised 80cm:** no = 0.0, 2A = .25, 1A = .50, light prompt = .75, unassisted = 1.0	6" beam	
**Category 5: Bear walk 16 feet on a 6 inch beam raised 80cm forwards:** no = 0.0, 2A = .25, 1A = .50, light prompt = .75, unassisted = 1.0	6" beam	
**Category 8: Walk sideways (L&R) on a 6 inch beam which elevated 80 cm off of the floor for 16 feet:** no = 0.0, 2A = .25, 1A = .50, light prompt = .75, unassisted = 1.0	6" beam	
**Category 10: Changing levels on a 6 inch beam elevated 80 cm off of the floor, from a sit, stand up, balance in relevee, reach down and pick up a beanbag, carry a beanbag:** no = 0.0, 2A = .25, 1A = .50, light prompt = .75, unassisted = 1.0	6" beam	
**Category 11: 5 beanbag tosses: Catch (2 hands) and throw underhand (L&R)with a beanbag tossed at random heights and directions while standing on a 6 inch beam elevated 80 cm off of the floor:** 1 or less catches = 0.0, 2 catches = .25, 3 catches = .50, 4 catches = .75, 5x consecutive catches = 1.0	6" beam	
**Category 13: Copies positions tuck, star, pike sit, butterfly and straddle stand on a 6 inch beam elevated 80 cm off of the floor:** 1 or less shapes = 0.0, 2 shapes= .25, 3 shapes= .50, 4 shapes = .75, 5 shapes= 1.0	6 " beam	
**Category 7: Walk forward on a 4 inch beam which elevated 20 cm. off of the floor for 16 feet:** no = 0.0, 2A = .25, 1A = .50, light prompt = .75, unassisted = 1.0	4" beam	
**Category 15: On the floor or tumbletrak,** Hopping forwards on the left foot 5x consecutively and then hopping on the right foot 5x consecutively: 0-2 x = 0.0, 3-4 x = .25, 5-6 x = .50, 7-8 x = .75, 9-10 x = 1.0	Floor	
**5**	**Apparatus**	**Score**
**Category 14: Jump sideways and backwards the length of the trampoline along the midline:** no = 0.0, 2A = .25, 1A = .50, light prompt = .75, unassisted = 1.0	trampoline	
**Category 16: Seat drop and stand up on the trampoline:** no = 0.0, entire skill assisted = .25, one phase assisted = .50, light prompt on one phase = .75, unassisted = 1.0	trampoline	
**Category 17: (a)Jumping over a series of 4 randomly spaced obstacles on the air track consecutively (b) 4 side to side ski jumps over an obstacle:** 4 or more extra bounces or pauses = 0.0, 3 or more extra bounces or pauses = .25, 2 or more extra bounces or pauses= .50, 1 or more extra bounces or pauses = .75, no extra bounces or pauses = 1.0	airtrack	

**Category 18: Climbs the rope on the air track:** no = 0.0, 2A = .25, 1A = .50, light prompt = .75, unassisted = 1.0	airtrack	
**Category 12: Jumping off a 40 cm height from 2 feet to land in balance on 2 feet:** no = 0.0, 2A = .25, 1A = .50, light prompt = .75, unassisted = 1.0	2 blocks	
**Category 19: Climbs up the wall bar 5 rungs using contralateral motionandthen climbs down 5 rungs:** no = 0.0, 2A = .25, 1A = .50, light prompt = .75, unassisted = 1.0	wall bar	
**Category 20:(a) Climb onto a 40cm height, (b) reach for and catch a trapeze or set of rings and swings (c) to land on 2 feet on a landing surface in balance with only verbal cues:** no = 0.0, 2A = .25, 1A = .50, light prompt = .75, unassisted = 1.0	trapeze	

6	Apparatus	Score
**Category 1: From a jump series (on air track, double mini trampoline) jump from 2 feet to an elevated incline mat to immediate dive roll:** no = 0.0, 2A = .25, 1A = .50, light prompt = .75, unassisted = 1.0	airtrack	
**Category 2: Execute a backwards roll down an incline mat with proper hand position to land on feet (phases: hand placement, rolling action, landing on feet):** no = 0.0, assist on 3 phases = .25, assist on 2 phases = .50, assist on 1 phase = .75, unassisted = 1.0	incline mat	
**Category 3: Crab walk forwards and crab walk backwards on a low set of parallel bars:** no = 0.0, 2A = .25, 1A = .50, light prompt = .75, unassisted = 1.0	P Bars	
**Category 8: Balance in a doggy position while rocking forwards and backwards and side to side on a teeter/pacman for 10 sec.:** no = 0.0, 2A = .25, 1A = .50, light prompt = .75, unassisted = 1.0	Pac Man	
**Category 7: While standing on a 4 inch beam, throw a beanbag underhand and hit a target 5 feet away with both the left hand and the right hand:** no = 0.0, 2A = .25, 1A = .50, light prompt = .75, unassisted = 1.0	4" beam	
**Category 9: : Walk backwards (feet must alternate and steps as long as the child's foot) on a 6 inch beam which is elevated 80cm for 16 feet:** no = 0.0, 2A = .25, 1A = .50, light prompt = .75, unassisted = 1.0	6" beam	
**Category 10: Walk 16 feet on a 4 inch beam elevated 80cm placing the feet heel to toe along a footprint pathway:** no = 0.0, 2A = .25, 1A = .50, light prompt = .75, unassisted = 1.0	4" beam	
**Category 11: Walk forward on a 4 inch beam elevated 80cm for 16 feet stepping over beanbags while carrying 2 shopping bags each filled with 6 beanbags:** no = 0.0, 2A = .25, 1A = .50, light prompt = .75, unassisted = 1.0	4" beam	
**Category 6: Crossing the midline. On a 6 inch beam, march 16 feet touching the opposite hand to knee:** no = 0.0, 2A = .25, 1A = .50, light prompt = .75, unassisted = 1.0	6" beam	

**Category 12: Walk sidewards on a 6 inch beam doing a forward grapevine with a left lead and also a right lead and then repeat with a backwards grapevine step:** no = 0.0, 2A = .25, 1A = .50, light prompt = .75, unassisted = 1.0	6" beam	
**Category 13: On a 6 " beam raised 80 cm hold a stork stand for 5 sec standing on left and then right foot:** no = 0.0, 2A = .25, 1A = .50, light prompt = .75, unassisted = 1.0	6" beam	
**Category 14: On the trampoline: (a)Seat drop,(b) doggy drop to (c)seat drop without standing up:** no = 0.0, assist on all phases = .25, assist on 1 phase= .50, light prompt on 1 phase = .75, unassisted = 1.0	trampoline	

6	Apparatus	Score
**Category 15: On the trampoline: Jump consecutively 10x in a series clockwise in the center boxes and then 10x counterclockwise:** 0-2 each = 0.0, 3-4 = .25, 5-6 = .50, 7-8 = .75, 9-10 = 1.0	trampoline	
**Category 16: Core strength: 10x assisted ant bounces on the trampoline consecutively:** 0-2 = 0.0, 3-4 = .25, 5-6 = .50, 7-8 = .75, 9-10 = 1.0	trampoline	
**Category 18: : On the trampoline using the center lines: Jumps consecutively 10 x side to side (L to R), and 10 x consecutively forward and backwards:** 0-2 each = 0.0, 3-4 = .25, 5-6 = .50, 7-8 = .75, 9-10 = 1.0	trampoline	
**Category 17: Hand/eye coordination: Holding a racquet, bat a balloon 5x consecutively and sit with a partner back to back on a ball and pass a ball behind your back and reach for it on the opposite side 5x and then standing back to back pass a ball overhead and between legs 5x:** 0-1 x = 0.0, 2 x = .25, 3 x = .50, 4 x = .75, 5 x = 1.0	floor (racquet: balloon, exercise ball)	
**Category 5: On the floor, hopscotch 8 steps forward with a L&R hop 8 steps:** < 2 = 0.0, 2-3 = .25, 4-5 = .50, 6-7 = .75, 8 = 1.0	floor	
**Category 19: Hold onto a bar and walk feet up the wall to an inverted position and touch belly button to the bar and hold for 5 seconds:** no = 0.0, 2A = .25, 1A = .50, light prompt = .75, unassisted = 1.0	single bar	
**Category 4: Bunny hop on the air track, tumbletrak or rod floor 10x consecutively:**0-2 each = 0.0, 3-4 = .25, 5-6 = .50, 7-8 = .75, 9-10 = 1.0	T trak	
**Category 20: Plyometrics: On the tumble trak jump up unto a block and immediately down off of the block 4x:** no = 0.0, 2A = .25, 1A = .50, light prompt = .75, unassisted = 1.0	T trak	

7	Apparatus	Score
**Category 2: 5x back extension raises, hands behind the head done consecutively:** 0-1x = 0.0, 2x = .25, 3x = .50, 4x = .75, 5x = 1.0	2 blocks	

**Category 3: Plyometrics: Using 3 blocks and one incline mat (on the rod floor): sequence up-down-up-down-up-down-dive roll with no extra bounces or pauses:** 4 or more extra bounces = 0.0, 3 extra bounces = .25, 2 extra bounces = .50, 1 extra bounce = .75, none = 1.0	3 blocks	
**Category 4: Walk feet up a wall to a 3/4 handstand position and do 5 wheelbarrow walks forward and 5 backwards:** 0-2 walks = 0.0, 3-4 walks = .25, 5-6 walks = .50, 7-8 walks = .75, 9-10 walks = 1.0	wall	
**Category 5: Cartwheel motion over a box:** no = 0.0, 2A = .25, 1A = .50, light prompt = .75, unassisted = 1.0	smartie and block	
**Category 6: In bear walk position on a low parallel bar turn 360˚ without touching the floor and using proper re-grasping sequence:** no = 0.0, 2A = .25, 1A = .50, light prompt = .75, unassisted = 1.0	P Bar	
**Category 7: Bunny hop forwards on a low set of parallel bars:** no = 0.0, 2A = .25, 1A = .50, light prompt = .75, unassisted = 1.0	P Bar	
**Category 8: Hold a front position on the parallel bars without touching the feet to the floor for 10 sec.:** 0-2 sec. = 0.0, 3-4sec. = .25, 5-6 sec. = .50, 7-8 sec. = .75, 9-10sec. = 1.0	P Bar	
**Category 1: Plank incurve hold, hands on the floor and hold for 10 sec.:** 0-2 sec. = 0.0, 3-4sec. = .25, 5-6 sec. = .50, 7-8 sec. = .75, 9-10sec. = 1.0	floor	
**Category 9: Long jump half of the body height using the proper arm swing 5x consecutively:** 1 = 0.0, 2 = .25, 3 = .50, 4 = .75, 5 = 1.0	Floor	
**Category 10: Marching on a 6" beam with contralateral arm action:** 0-2 steps = 0.0, 3-4 steps = .25, 5-6 steps = .50, 7-8 steps = .75, 9-10 steps = 1.0	floor	
**Category 11: Skipping on the ground 10 steps using contralateral arms and legs:** no = 0.0, using the footprints (legs only) = .25, footprints and leg and arm action = .50, legs only = .75, arms and legs = 1.0	floor	
**Category 14: On a 4 " beam raised 100 cm off of the floor do a forward grapevine with a left lead and a right lead for 16 feet each. Repeat with a backward grapevine with a left lead and a right lead for 16 feet each:** no = 0.0, 2A = .25, 1A = .50, light prompt = .75, unassisted = 1.0	4" beam	
**Category 12: Walk forward on a 6 inch beam while throwing and catching a beanbag in the air 10x without stops or pauses:** 0-2 steps = 0.0, 3-4 steps = .25, 5-6 steps = .50, 7-8 steps = .75, 9-10 steps = 1.0	6" beam	
**Category 13: Crossing midline: Walk on a 6 inch beam and pick up a beanbag with the left hand and cross the midline to place it in a bucket on the right side of the beam. Repeat with the right hand. Repeat 6x:** no = 0.0, 2A = .25, 1A = .50, light prompt = .75, unassisted = 1.0	6" beam	
7	Apparatus	Score
**Category 15: On a 6" beam raised 100 cm off of the floor do alternating grapevine steps forwards and backwards with a left foot lead and then a right foot lead:** no = 0.0, 2A = .25, 1A = .50, light prompt = .75, unassisted = 1.0	6" beam	

**Category 16: On the trampoline with a skipping rope, skip 10x consecutively:** 0-2x = 0.0, 3-4x = .25, 5-6x = .50, 7-8x = .75, 9-10x = 1.0	Trampoline	
**Category 17: 4 skill sequence on the trampoline : skill choices (seat , doggy or stomach drop) shape jumps (full turn, tuck or star jump). The routine must contain 2 skill jumps:** 4 or more extra bounces = 0.0, 3 extra bounces = .25, 2 extra bounces = .50, 1 extra bounce = .75, none = 1.0	Trampoline	
**Category 20: 5x unassisted ant bounces in a series with no extra bounces:** 0-1x = 0.0, 2x = .25, 3x = .50, 4x = .75, 5x = 1.0	Trampoline	
**Category 18: Climbs onto a knotted rope and maintains balance for 10 sec.:** 0-2 sec. = 0.0, 3-4sec. = .25, 5-6 sec. = .50, 7-8 sec. = .75, 9-10sec. = 1.0	Knotted rope	
**Category 19: Steers, pedals and brakes a bike with training wheels 40 feet:** no = 0.0, 2A = .25, 1A = .50, light prompt = .75, unassisted = 1.0	Bike riding	

8	Apparatus	Score
**Category 1: Inversion: On a single bar do a skin the cat:** no = 0.0, 2A = .25, 1A = .50, light prompt = .75, unassisted = 1.0	Bar	
**Category 2: Inversion: Front support and forward tuck roll down on a single bar:** no = 0.0, 2A = .25, 1A = .50, light prompt = .75, unassisted = 1.0	Bar	
**Category 19: Stabilizer muscles: Front support on a single bar and cast 5x:** 0-1 x = 0.0, 2 x = .25, 3 x = .50, 4 x = .75, 5 x = 1.0	Bar	
**Category 3: Locomotion: On the floor chasse forward with a left foot lead for 10 steps, repeat with a right foot lead:** 0-2 steps = 0.0, 3-4 steps = .25 ,5-6 steps = .50, 7-8 steps = .75, 9-10 steps = 1.0	Floor	
**Category 4: On a smartie mat perform 3 steps to an assemble and jump to a landing 5x consecutively:** 0-1 x = 0.0, 2 x = .25, 3 x = .50, 4 x = .75, 5 x = 1.0	floor (smartie mat)	
**Category 5: Coordination: Inchworm: 4 steps forward with hands followed by 4 steps forward with feet 5x consecutively:** 0-1 x = 0.0, 2 x = .25, 3 x = .50, 4 x = .75, 5 x = 1.0	Floor	
**Category 18: Eye hand coordination: Throw and catch 1 beanbag from one hand to the other 10x consecutively:** 0-2 x = 0.0, 3-4 x = .25, 5-6 x = .50, 7-8 x = .75, 9-10 x = 1.0	Floor	
**Category 7 is: Sprint a minimum of 20 metres with contralateral arm action:** 0-4m = 0.0, 5-9m = .25, 10-14m = .50, 15-19m = .75, 20m+ = 1.0	floor	
**Category 6 is: Crawling 16 feet on a 6 inch beam carrying a beanbag underneath your chin:** no = 0.0, 2A = .25, 1A = .50, light prompt = .75, unassisted = 1.0	6" Beam	
**Category 8: While standing on a 6 inch beam raised 100 cm off the ground jump over 6 beanbags placed in a row:** 2 bags = 0.0, 3 bags = .25, 4 bags = .50, 5 bags = .75, 6 bags = 1.0	6" Beam	

**Category 9: Motor planning: While standing on a 6 inch beam raised 100 cm off the ground jump over a series of 6 beanbags placed in a row without stopping:** 2 bags = 0.0, 3 bags = .25, 4 bags = .50, 5 bags = .75, 6 bags = 1.0	6" beam	
**Category 11: Midline: While sitting on a 6 inch beam in a pike position pass a beanbag clockwise behind your back and pass it to the other hand and return it to the front of the body repeat counterclockwise:** no = 0.0, 2A = .25, 1A = .50, light prompt = .75, unassisted = 1.0	6" Beam	
**Category 20: Core strength: 10x tuck sit ups on a 6 inch beam (beanbag in both hands and touch the beam, the therapist may hold the ankles, beanbag must touch the therapists shoulder):** 0-2 x = 0.0, 3-4 x = .25, 5-6 x = .50, 7-8 x = .75, 9-10 x = 1.0	6" Beam	
8	Apparatus	Score
**Category 10: Motor planning: While standing on a 4 inch beam raised 100 cm off the ground step over a series of 6 beanbags placed in a row without stopping while describing the beanbag (i.e.. color/design):** 2 bags = 0.0, 3 bags = .25, 4 bags = .50, 5 bags = .75, 6 bags = 1.0	4" beam	
**Category 12: Walk sidewards on a 4 inch beam doing a grapevine alternating forward and backward stepping for 16 feet with a left lead. Repeat with a right lead:** no = 0.0, 2A = .25, 1A = .50, light prompt = .75, unassisted = 1.0	4" beam	
**Category 13: 2 different 5 skill routines with 3 skills on the trampoline: Skill choices (seat , doggy , stomach or back drop, swivel hips) Shape jumps (full turn, tuck or star jump):** 4 or more extra bounces = 0.0, 3 extra bounces = .25, 2 extra bounces = .50, 1 extra bounce = .75, none = 1.0	trampoline	
**Category 14: On the trampoline perform a back drop from feet to feet:** feet to back from a stand with a crash mat= 0.0, from back to feet = .25, from feet to back with a bounce = .50, from feet to feet with assistance (PEC or copy)= .75, feet to feet unassisted with a bounce=1.0	trampoline	
**Category 15: On the trampoline sequence: feet to doggy to 3/4 forward salto to feet:** from doggy to back with assistance = 0.0, from a stand with assistance = .25, from doggy to a crash = .50, from a stand to doggy to back unassisted = .75, from feet to doggy to feet= 1.0	trampoline	
**Category 16: Skills on the trampoline: Longitudinal twisting skill (swivel hip, back drop 1/2 to feet, barrel roll):** 1 person assist = 0.0, with a throw mat = .25, with a pic symbol = .50, copying = .75, unassisted = 1.0	trampoline	
**Category 17: Skills on the trampoline: 10 consecutive bounces on the trampoline using proper arm action and increasing height with each bounce:** 0-2 x = 0.0, 3-4 x = .25, 5-6 x = .50, 7-8 x = .75, 9-10 x = 1.0	trampoline	
