# Supplementary material for: Gymnastic-Based Movement Therapy for Children With Neurodevelopmental Disabilities: Results From a Pilot Feasibility Study
Source: Front Pediatr. 2019 May 14;7:186. doi: 10.3389/fped.2019.00186 (PMC6527593; doi:10.3389/fped.2019.00186)
Supplement: S2 Appendix — ESMT scientific protocol: visual representation. [file Data_Sheet_2.PDF]

EMPOWERING STEPS® MOVEMENT THERAPY

© 2008 Symington Teaching Programs Inc.

Scientific Protocol: Visual Representation[V2]

Written by: Vivien Symington B.A./B.P.H.E

March 2017

Acknowledgements: Caroline Standring M.A./P.G.C.E., Kristyn Jorgenson BSc. Bio, Garrick Mah BSc. Kin, John Oh

EMPOWERING STEPS® MOVEMENT THERAPY
© 2008 Symington Teaching Programs Inc.

Introduction

The Empowering Steps Movement Therapy (ESMT) program is a child centered holistic motor intervention program that focuses on function rather than diagnosis, treating all children with neurodevelopmental disabilities.

The ESMT process focuses on three main components:

- Motor Development
- Emotional State
- Attachment

The motor activities act as a medium allowing the therapist to focus on affecting a change to the child's emotional state and ability to develop attachment. Therapists are trained to adapt to the child's needs on a daily basis in order to reduce fear, anxiety, and stress levels while building a trusting relationship. This results in the child becoming more emotionally regulated, trusting, cooperative and physically capable. At this point the therapist encourages social interaction with other ESMT children and therapists attending at the same time. The ultimate goal is to fully integrate ESMT children into neurotyical classes or specialized ESMT groups which focus primarily on social development.

ESMT is a highly structured program that promotes each child's development by setting individualized goals. Progress is charted daily, and two formal assessments are conducted each year. ESMT focuses on all aspects of development to achieve and maximize each child's potential.

ESMT uses this combination to target the implicit memory system. Implicit memory is learning without awareness (Kleynen et al., 2015). By focusing on implicit memory we strive to affect the neurobiology of each child without them having to be conscious participants (Siegel, 2007; Stillman et al., 2016). Memory influences a child's behaviour, emotional state, perception, physical development and motor development(Siegel, 2007; Stillman et al., 2016).

© 2008 Symington Teaching Programs Inc.

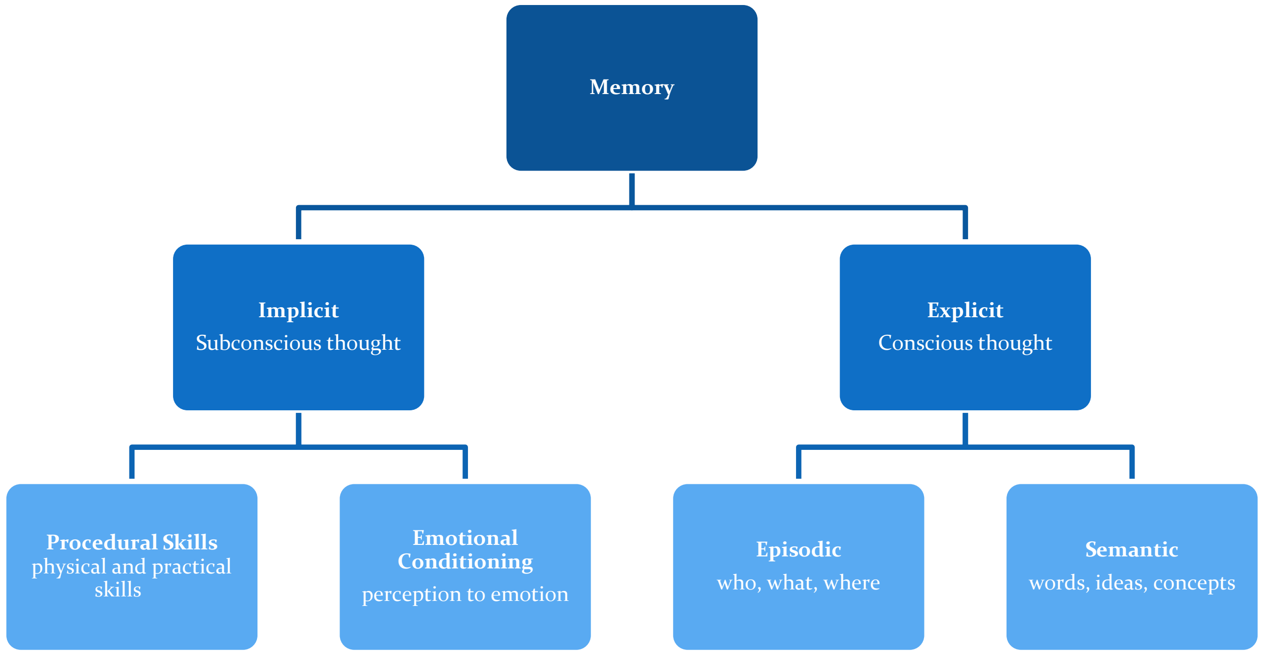

Figure 1.ESMT uses implicit learning techniques to develop learning with trial and error, repetition and discovery learning using scaffolding techniques which ensure the child is always in their zone of proximal development (Bodrova & Leong, 1996). This leads to explicit memory, internalizing success, and self efficacy. "For the first year or life, the infant has available an "implicit" form of memory that includes emotional, behavioural, perceptual, and perhaps bodily (somatosensory) forms of memory (Siegel, 2007)."

© 2008 Symington Teaching Programs Inc.

Goal

- **Research Validation:** Leading to accreditation of ESMT as one of the best practice therapeutic interventions for children with neurodevelopmental disabilities.

Objectives

- **Motor Development:** To facilitate a child's learning of gross and fine motor skills in order to help them achieve his/her highest potential in functional motor capabilities. Upon completion of the ESMT motor scale, a child is capable of executing motor skills that correspond to the developmental milestones of a neurotypical child 12 years of age.
- **Emotional State:** To affect a change in the way a child views a specific event in their memory system. We know that many children with Neurodevelopmental Disabilities (NDD) have a great deal of fear and anxiety and that stress is toxic to the brain and its development. Our focus is on rebuilding the emotional state of each child to one filled with curiosity, discovery and joy.
- **Attachment**: To provide an inclusive environment which helps each child to develop trusting and caring relationships with their therapist, family and community helping them to engage and integrate joyfully into their community as a contributing participant.

© 2008 Symington Teaching Programs Inc.

ESMT PROCESS: During a child's intake process they are assessed on their emotional state, motor development and attachment. Each of these areas is given equal focus and attention and deficits in each area form part of each child's personalized plan.

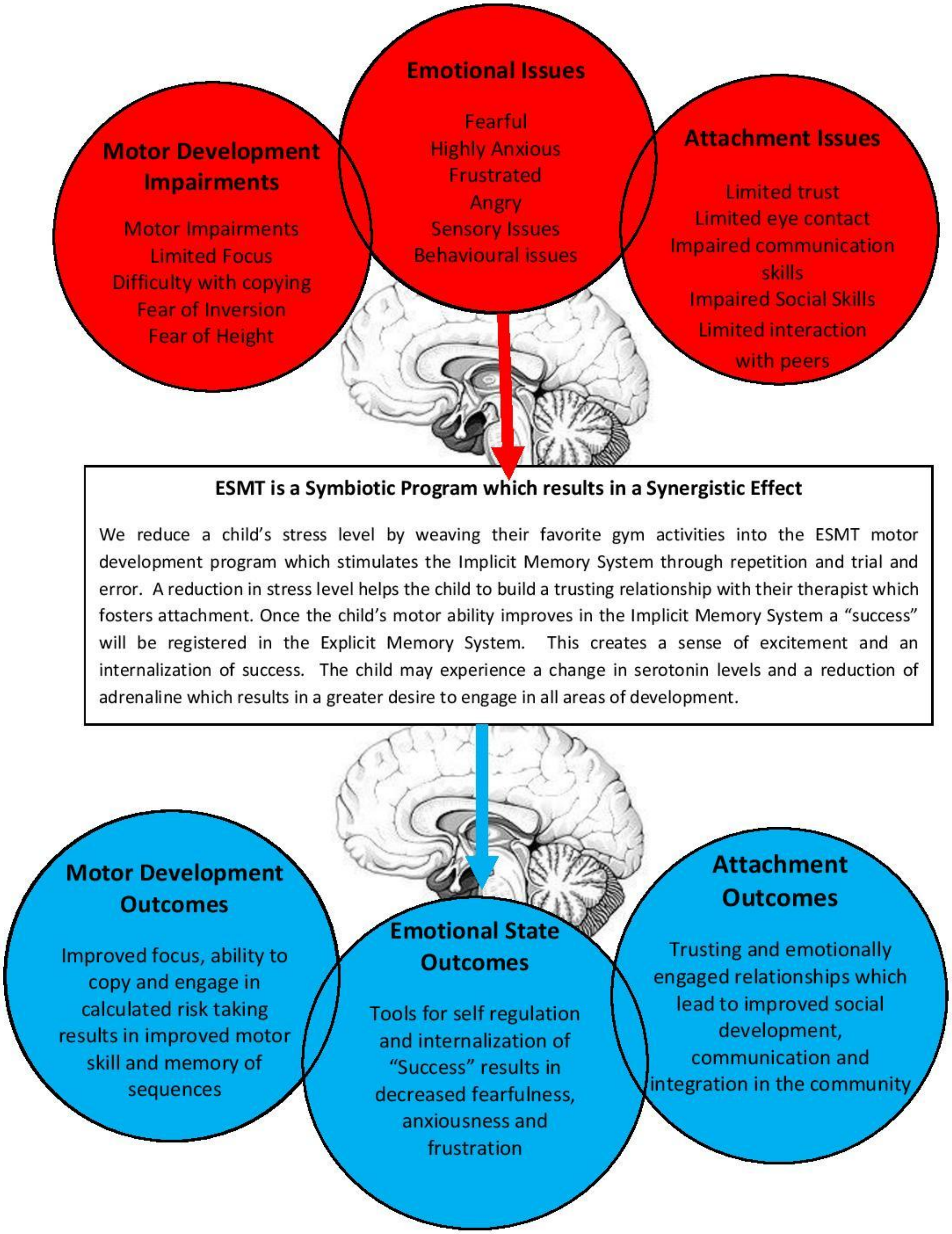

Figure 2.

EMPOWERING STEPS® MOVEMENT THERAPY

© 2008 Symington Teaching Programs Inc.

References:

Kleynen, M., Braun, S. M., Rasquin, S. M. C., Bleijlevens, M. H. C., Lexis, M. A. S., Halfens, J., … Beurskens, A. J. (2015). Multidisciplinary views on applying explicit and implicit motor learning in practice: An international survey. *PLoS ONE*, *10*(8), 1–15. https://doi.org/10.1371/journal.pone.0135522

Siegel, D. (2007). Toward an interpersonal neurobiology \nof the developing mind: \nAttachment relationships, “mindsight,” \nand neural integration. *Infant Mental Health Journal*, *22*, 67–94. https://doi.org/10.1002/1097-0355(200101/04)22:1<67::AID-IMHJ3>3.0.CO;2-G

Stillman, C. M., Watt, J. C., Grove, G. A., Wollam, M. E., Uyar, F., Mataro, M., … Erickson, K. I. (2016). Physical activity is associated with reduced implicit learning but enhanced relational memory and executive functioning in young adults. *PLoS ONE*, *11*(9), 1–21. https://doi.org/10.1371/journal.pone.0162100

Bodrova, E., & Leong, D. (1996). *Tools of the mind: The Vygotskian approach to early childhood education*. Englewood Cliffs, N.J: Merrill.

Literature Reviewed:

Aquilla, P. (2006). Sensory processing disorder: A summary of the current science behind sensory processing. *INspire! 1(1),* MukiBaum Treatment Centres.

Boon, R. (n.d.) *Neurodevelopmental therapy (Inhibition of primitive reflexes).* Sydney, AUS: Learning Discoveries Psychological Services. Retrieved April 11, 2007, from http://home.iprimus.com.au/rboon/NeurodevelopmentalTherapy.htm

Cohen, B. B. (1993). *Sensing, feeling, and action: The experiential anatomy of body-mind centering.* Northampton, MA: Contact Editions.

Doidge, N. (2007). *The brain that changes itself: Stories of personal triumph from the frontiers of brain science.* New York, New York: Viking Penguin, Penguin Group (USA) Inc.

Doman, G. (1990). *What to do about your brain-injured child*. Philadelphia, PA: The Better Baby Press.

Elliott R. O., Jr., Dobbin, A. R., Rose, G. D., & Soper, H. V. (1994). Vigorous, aerobic exercise versus general motor training activities: Effects on maladaptive and stereotypic behaviors of adults with both autism and mental retardation. *Journal of Autism and Developmental Disorders, 24,* 565-576.

Frith, U. (1989). *Autism: Explaining the enigma*. Oxford: Blackwell.

Gardner, H. (1983). *Frames of mind: The theory of multiple intelligences*. New York: Basic Books.

Green, N.S. (2003). *The Adventure Trails* [ebook]. SanDiego, CA: KW Publishing.

© 2008 Symington Teaching Programs Inc.

Greenspan, S.L. & Wieder, S. (2003). *Engaging autism: The floortime approach to helping children relate, communicate and think*. Jackson, TN: Perseus Books.

Hearing, Speech and Deafness Centre: *Information on Autism*. Retrieved February 21, 2008, from http://www.hsdc.org:80/Child/Speech/autismdefine.htm

Hill, D.A. & Leary, M. R. (1993). *Movement disturbance: A clue to hidden competencies in persons diagnosed with autism and other developmental disabilities* Madison, Wisconsin: DRI Press.

Hogan, K. (2004). *The Ear and the Alexander Technique*. Retrieved February 21, 2008, from: http://www.kayhogan.com/articles_ear.htm

Kanner, L. (1973). *Childhood Psychosis: Initial studies and new insights.* New York: Wiley.

Kaufman, S. L. (1998). The Son-Rise Program at the Option Institute. *Communication*, Spring 1998, pp19 IV 23.

Kodiak, E. (2006). *Rappin' on the reflexes: A practical guide to infant reflexes*. Temple, NH: Sixth Printing.

Kolb, D. A. (1984). *Experiential learning: Experience as the source of learning and development.* New Jersey: Prentice-Hall.

Louv, R. (2006). *Last child in the woods: Saving our children from nature-deficit disorder*. Chapel Hill, NC: Algonquin Books.

Schmitz, C., Martineau, J., Barthelemy, C., Assaiante, C. (2003). Motor control and children with autism: deficit of anticipatory function? *Neuroscience Letters, 348*(1), 17-20.

Vernazza-Martin, S., Martin, N., Vernazza, A., Lepellec-Muller, A., Rufo, M., Massion, J., & Assaiante, C. (2005). Goal directed locomotion and balance control in autistic children. *Journal of Autism and Developmental Disorders, 35*(1), 91-102.

Wilbarger, P., & Wilbarger, J. (1991). *Sensory defensiveness in children aged 2-12: An intervention guide for parent and other caretakers*. Denver, CO: Avanti Educational Programs.

Williamson, G, G., & Anzalone, M.E., (2001). *Sensory integration and self -regulation in infants and toddlers: Helping very young children interact with their environment.* Washington, DC: Zero To Three.

Yack, E., Sutton, S., & Aquilla, P. (1998). *Building bridges through sensory integration.* Las Vegas, Nevada: Sensory Resources.

© 2008 Symington Teaching Programs Inc.
