# Supplementary material for: Gymnastic-Based Movement Therapy for Children With Neurodevelopmental Disabilities: Results From a Pilot Feasibility Study
Source: Front Pediatr. 2019 May 14;7:186. doi: 10.3389/fped.2019.00186 (PMC6527593; doi:10.3389/fped.2019.00186)
Supplement: S3 Appendix — Overview of the ESMT program. [file Data_Sheet_3.PDF]

EMPOWERING STEPS®

© Symington Teaching Programs Inc. 2008

Overview of the ESMT Program

Written by: Vivien Symington B.A/B.P.H.E

November 2018, Version 4

Acknowledgements: Kristyn Jorgenson B.Sc Bio, Alison Davidson B.Sc Kin, Garrick Mah B.Sc Kin,

EMPOWERING STEPS®

© Symington Teaching Programs Inc. 2008

Overview of the ESMT Program

Introduction

The ESMT program is comprised of 10 stages of motor development, each encompassing 20 motor skills. When a child begins the program, they are assessed on their current motor function and placed on a corresponding stage. As their motor skills develop, they are introduced to more complex skills and make their way into the higher stages. Assessments are completed semi-annually and used in a prescriptive manner to ensure that each child is still adequately challenged and working within their zone of proximal development. Although several children may be working on the same ESMT stage of motor development, the program is adapted to each child's individual needs. The therapists acknowledge each child's unique learning styles, weaknesses and strengths to maximize their potential for success. The ideal goal of the program is to achieve a level of motor development as close as possible to their peers, so that they feel more confident and experience inclusivity in a greater variety of settings.

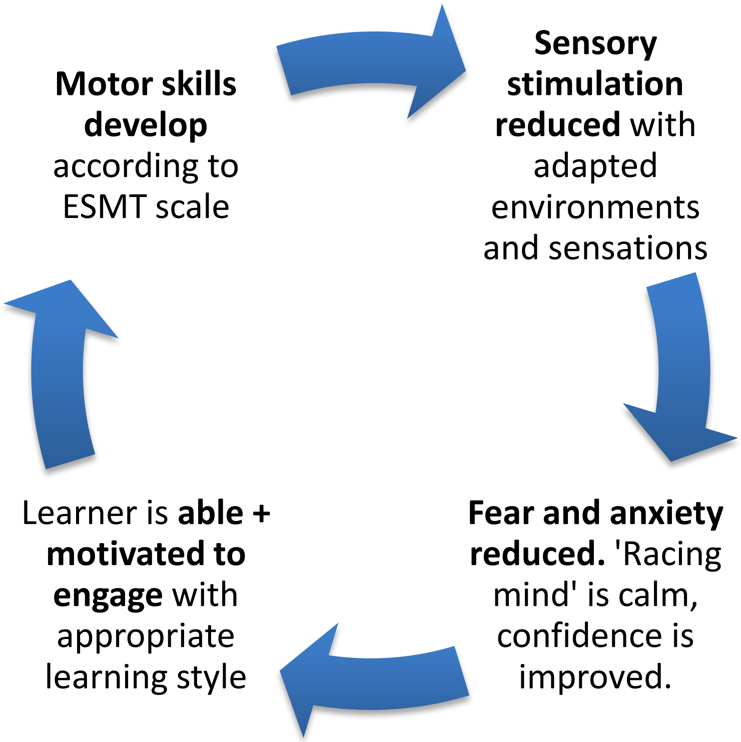

The ESMT motor scale was developed to measure the gross motor function of children with physical and cognitive disabilities. Neuro-typical children, in most cases, will learn how to crawl, walk, jump, climb, descend stairs with alternating feet and run with contra-lateral arm action naturally. Often this is not the case for children with a physical or cognitive disability. They must be taught through a repetitive and consistent intervention which is broken down and scaffolded into manageable motor skill components. For example, before a child crawls, he or she must have enough shoulder girdle strength

© Symington Teaching Programs Inc. 2008

to maintain a doggy (hand and knee) position. Therefore, before a child is taught to crawl they are first taught how to maintain a doggy position and then how to shift their weight from one arm to the other while maintaining the position.

Additionally, many children with a cognitive or physical disability have difficulty jumping from two feet. If a child cannot land safely on two feet with bent knees, they limit their ability to climb, walk on uneven surfaces and explore their environment. This leads to high anxiety and an unwillingness to participate in physical activities. The ESMT motor scale assesses and prescribes a program for teaching a child to jump from 2 feet and to land on 2 feet.

ESMT Motor Scale

The ESMT motor scale is unique because it is function-based, and can be applied to children of all ages and diagnoses. The ESMT motor scale has 10 stages of motor development. All skills are carefully scaffolded from one stage to the next ensuring that each child is constantly working within their zone of proximal development which increases their motivation for goal attainment. All children, irrespective of age or diagnosis will possess a certain level of motor function that can be found somewhere throughout the 10 stages. Following the initial assessment, each child is placed on the motor scale according to their current motor function. For example, a non ambulatory 12 year old child with cerebral palsy may be placed on stage 1, 2, or 3 depending on functionality, whereas an ambulatory 3 year old child with autism may be placed on stage 4 or 5. Developmental milestones that may be missing are also identified and incorporated into their sessions.

During the semi-annual assessment, an ESMT Supervisor observes the child's session and uses the ESMT motor scale V.2(Appendix 3) to quantitatively assess motor improvements. Qualitative observations are also noted regarding biomechanics, muscle imbalances, attachment, emotional state, co-regulation and self-regulation strategies, social interaction and current methods of program delivery (picture/written schedules, cooperative, circuit, exploratory, etc.). The supervisor consults with the child's regular ESMT therapist and parent(s) to gather any other relevant information. The ESMT supervisor then compiles this information into a comprehensive report, in addition to providing goal revisions and any suggested changes to the program delivery. The semi-annual report will form the prescription for the next 5 month period.

The ESMT model recognizes that not all children develop at the same rate. ESMT therapists are trained to adapt to each individual child's unique needs so that they can successfully complete the gross motor skill requirements to their best ability.

Sessions

Empowering Steps Movement Therapy (ESMT) sessions
The 1:1 nature of ESMT sessions allows the therapist to approach each child as an individual and tailor their program specifically to meet their functional, social, and emotional needs according to their

© Symington Teaching Programs Inc. 2008

learning style. This process helps to foster attachment with the therapist, reduce fear and anxiety associated with new or difficult skills and encourage exploration and participation in the ESMT environment. As the child becomes a more willing participant and begins to attempt new skills with increased confidence, the therapist is able to focus more attention on the child's social interaction with other children in the gym. Encouraging the child to engage with his/her peers during their session facilitates an improvement in social confidence and integration with another individual or group may be considered. The integration process involves learning to participate in an instructional gymnastics class with the support of their therapist, during which social interaction and class etiquette (following directions, staying with the class, turn taking, verbal interaction with peers) are emphasized.

Therapists

In order to become an ESMT therapist, an interested candidate must first attend a 2 hour orientation on the ESMT program. Following the orientation, a weekly schedule is assigned to each potential trainee to assist with children's sessions. If a potential trainee is ranked as a candidate for training, they are invited to enter the ESMT therapist training program. This program requires all potential staff members to train under an experienced ESMT supervisor for a minimum of 480 hours. Not all trainees become ESMT therapists. Trainees must meet the ESMT standard of care and gain the confidence of the ESMT clients, colleagues and management. Once the candidate has met these criteria they are assessed as follows: 1) real time evaluation of sessions with 3 different children they were assigned to throughout the training period; 2) successful completion of the ESMT Theory Modules and written exam.

Environment

Most children love the environment provided by gymnastics facilities. This environment is rich with the types of activities a neuro-typical child loves to explore such as jumping on trampolines, playing in a foam pit, climbing apparatus, balancing on beams and swinging on bars. The ESMT motor scale was designed to develop the ability of a child with a cognitive or physical disability to participate fully in this environment.

Children in the ESMT program are exposed to many other children participating in programs while they are having their session. Programs can range from classes for children 1 year of age to adults. The ability level of each class can range from an introductory program (usually 1 hour/week) to a high performance athlete training for National and International competition (up to 25 hours/week).

Children in the ESMT program participate symbiotically with other children throughout the gym. ESMT children are often at an apparatus for 2 – 5 minutes at a time before moving to a new station. Sometimes they will be alone on a station, for example when they are in a swinging apparatus, and other times they are sharing a station with a class for 2-3 minutes. A typical class spends 15 minutes on each station.

All children in the gym are applauded for effort and not skill level. When a child on the competitive team achieves a skill which is in their zone of proximal development, everyone stops to watch and

© Symington Teaching Programs Inc. 2008

applaud. Similarly, when an ESMT child achieves a milestone in their zone of proximal development, such as a child with Cerebral Palsy jumping independently on the trampoline for the first time, everyone stops to watch and applaud. It is often more moving to witness a breakthrough from a child living with a cognitive or physical disability. The ESMT model fosters a culture that acknowledges perseverance, effort, courage, and trust irrespective of any child's individual circumstances.

Other Programs

ESMT Respite Camps

ESMT Respite camp programs may be offered during school holidays such as winter, spring, and summer breaks. ESMT Respite Camps focus on social integration, physical development, and communication skills. Activities include ESMT, games, arts and crafts, free time, hiking, bike riding, and ESAT. Similar to a typical session, each child is paired with an ESMT therapist. If a child has the ability to follow instructions and interact with others, he or she may be paired with another child of similar motor and cognitive ability to foster relationship building among peers. The ESMT Respite Camps run symbiotically with typical gymnastic camps providing an opportunity for children enrolled in the ESMT Respite camp to intermingle with peers of all abilities.

ESMT Life Skills Camps

ESMT Life skills camps are offered in the summer for groups of 4 or more children and focus on social interaction while navigating common day to day activities such as grocery shopping, cooking, public transit, nature hikes, public parks, playgrounds and other fun, educational venues. Throughout the camp, the children are encouraged to participate in team activities such as scavenger hunts, relays, field sports, and games.

Empowering Steps Aqua Therapy (ESAT) sessions

The ESAT program is a one-to-one therapy program that encompasses ESMT skills, swimming skills, safety skills, and physical conditioning in a fun and wet environment. Similar to the ESMT program, every participant is assessed and placed in the stage (1-10) reflecting their current zone of proximal development. Skill progressions range from getting into the water comfortably to stroke development and jumping off the diving boards. ESAT uses a similar model to the ESMT program focusing on each child's abilities rather than their diagnosis. There is a strong emphasis on building a trusting relationship with the therapist in the water.

Social Development

Special Events

In 2008, the West Coast Movement Therapy Society (WCMTS) was established by ESMT family members and friends, to support the children and their families enrolled in the ESMT program. The society with the support of Vivien Symington offers special family events throughout the year to enhance social development and a sense of community and belonging. Throughout the year the WCMTS organizes the following events: Christmas dinner with Santa, Bowling Night, Year End show, and the annual Symington Endowment Fund 5k Roll, Walk, Run. These events encourage family bonding, networking,

© Symington Teaching Programs Inc. 2008

and inclusion. The WCMTS also covers the cost of an ESMT therapist to assist children enrolled in the ESMT program to host or attend birthday parties at Club Aviva. The WCMTS believes that this type of support encourages social interaction between ESMT children and their peers while modeling an inclusive environment for children of all abilities.

Following is a detailed description of one of these events and how it enhances community inclusion for ESMT families. Dinner with Santa originate because many children in the ESMT program had traumatic mall experience when they were taken to visit Santa. It has since become a very popular annual event with 70 plus people in attendance. Not only does Santa attend for picture taking and gift giving, pizza is provided by the WCMTS and caroling is led by ESMT staff members. Following dinner, an hour of gym drop in is provided free of charge by the WCMTS so that parents can leave their children at the gym and do some Christmas shopping.

In 2009, Vivien Symington founded the Symington Endowment Fund which is held by the Coquitlam Foundation. This fund was established to empower children with cognitive and physical disabilities, youth-at-risk, and high performance gymnasts to access programs encouraging healthy, physical, and emotional development. The fund provides financial assistance in the form of grants and bursaries. Children in the ESMT program are eligible to apply for a bursary to attend summer respite camp.

In 2018, Vivien Symington founded the Symington Symbiotic Foundation which is the umbrella organization for the expansion of the ESMT program.

© Symington Teaching Programs Inc. 2008
